# Subdiffusive-Brownian crossover in membrane proteins: a generalized Langevin equation-based approach

**DOI:** 10.1016/j.bpj.2021.09.033

**Published:** 2021-09-28

**Authors:** Loris Di Cairano, Benjamin Stamm, Vania Calandrini

**Affiliations:** 1 Department of Physics, Faculty of Mathematics, Computer Science and Natural Sciences, Aachen University, Aachen, Germany; 2 Applied and Computational Mathematics, Department of Mathematics, RWTH Aachen University, Aachen, Germany; 3 Computational Biomedicine, Institute of Neuroscience and Medicine INM-9 and Institute for Advanced Simulations IAS-5, Forschungszentrum Jülich, Jülich, Germany

## Abstract

In this work, we propose a generalized Langevin equation-based model to describe the lateral diffusion of a protein in a lipid bilayer. The memory kernel is represented in terms of a viscous (instantaneous) and an elastic (noninstantaneous) component modeled through a Dirac *δ* function and a three-parameter Mittag-Leffler type function, respectively. By imposing a specific relationship between the parameters of the three-parameter Mittag-Leffler function, the different dynamical regimes—namely ballistic, subdiffusive, and Brownian, as well as the crossover from one regime to another—are retrieved. Within this approach, the transition time from the ballistic to the subdiffusive regime and the spectrum of relaxation times underlying the transition from the subdiffusive to the Brownian regime are given. The reliability of the model is tested by comparing the mean-square displacement derived in the framework of this model and the mean-square displacement of a protein diffusing in a membrane calculated through molecular dynamics simulations.

## Significance

This study reports a generalized Langevin equation model, based on a three-parameter Mittag-Leffler memory kernel, to describe a protein laterally diffusing in a membrane. The model captures the different dynamical regimes, namely ballistic, subdiffusive, and Brownian, as well as the crossover between them. The spectrum of relaxation times underlying the transition from the subdiffusive to the Brownian regime is given.

## Introduction

Lateral diffusion in membrane is key for cellular information processing (1). Cell membrane fluidity determines lipid and protein mixing, thus regulating diffusion-limited biochemical interaction rates responsible for signal transduction from the extracellular to the intracellular environment.

In the last few years, it has been studied how the biological structures and features of living cells, such as membrane composition, concentration of proteins in membrane, compartmentalization, and crowding, affect the diffusion of proteins and lipids in membrane (2,3). A plethora of experimental studies in cellular membranes (4, 5, 6, 7, 8) and in vitro lipid bilayers (9, 10, 11), as well as computer simulations of minimalistic model membranes in crowding conditions (12, 13, 14, 15, 16, 17), have shown a deviation from the simple Brownian diffusion, in which the random displacements are described by a Gaussian probability distribution and the mean-square displacement (MSD) increases linearly in time when time lags much longer than the typical collision time are considered (*MSD* ∝ *Dt*, where *D* is the diffusion constant, *D* = *k*_*B*_*T*/*μ*, with *k*_*B*_, *T*, and *μ* being the Boltzmann constant, the temperature, and a constant accounting for the geometry of the particle and the kinematic viscosity of the environment, respectively). One of the most familiar phenomena is indeed a sublinear, power-law increase of the *MSD* ∝ *D*_*α*_*t*^*α*^, with 0 < *α* < 1 and *D*_*α*_ representing a diffusion constant with the physical dimensions of [*L*^2^/*t*^*α*^].

Such “subdiffusive” dynamics, also referred to as anomalous diffusion, is commonly attributed to the densely packed and heterogeneous structures resulting from the crowding of the biological membranes as discussed in (12,18). Notably, atomistic and coarse-grained simulations, as well as this study, have also shown subdiffusive behavior for lipids and proteins in simple lipid bilayers in the limit of infinite protein dilution (19, 20, 21). Moreover, as observed in many viscoelastic systems, subdiffusivity is a transient dynamical behavior (22). After the short-time ballistic regime, in which the tagged particle freely diffuses (*MSD* ∝ (*k*_*B*_*T*/*M*)*t*^2^, with *M* being the particle’s mass), the interactions with the medium and its specific fluidic and mechanical properties can lead to nontrivial persistent correlations responsible for the subdiffusive behavior (23). Yet, for time lags exceeding some characteristic time, the standard Brownian dynamics is recovered. This Brownian regime is well described by the celebrated Saffman and Delbrück model (24,25) and its extensions (26, 27, 28, 29, 30), and a logarithmic dependence of the diffusion coefficient as a function of the protein radius (*D* ∝ log(1/*R*)) is found. Nevertheless, in protein-crowded membranes, a deviation from the Saffman and Delbrück model law has been shown (31), and one finds *D* ∝ 1/*R*. The crossover from the subdiffusive to the standard Brownian dynamics can take place over a quite large time window, and the transition onset strongly depends on packing and crowding, ranging from a tenth to hundreds of nanoseconds for lipids in protein-free membranes or proteins in membranes at infinite protein dilution up to arbitrarily long timescales for crowded real systems (20,22,32). As a consequence, diffusive properties can no longer be characterized by a single diffusion constant or by a single exponent *α*.

Several theoretical frameworks have been introduced to reproduce the subdiffusive behavior and describe the physical mechanism behind it (see, for instance, (22) and references therein for a complete overview on their theoretical foundations and applications to biological systems). Among them, we recall the so-called Gaussian models such as the fractional Brownian motion (FBM) (33) and the generalized Langevin equation (GLE) (22), in which the statistics of the noise in the relevant stochastic equations is still assumed to be Gaussian, as in the Langevin approach for normal Brownian diffusion, but, differently from this, the noise displays persistent power-law correlations (power-law Gaussian noise), leading to a sublinear increase of the MSD.

The FBM is defined as a zero mean Gaussian process *B*^(*H*)^(*t*), where *H* is in the interval (0, 1) and its autocorrelation function is $\left\langle B^{\left(H\right)}\left(t\right)B^{\left(H\right)}\left(\tau \right)\right\rangle$ = (|*t*|^2*H*^ + |*τ*|^2*H*^ − |*t* − *τ*|^2*H*^)/2 for any *t*, *τ* ≥ 0 (22,34,35). Because the FBM is a generalization of the Brownian motion, which is obtained for *H* = 1/2, it is in general a non-Markovian process. The FBM process has two properties: self-similarity and stationary increments. Self-similarity means that the time segment of the FBM trajectory has the same behavior as any segments of other timescales after an appropriate normalization. Second, stationary increment means that the distribution of *B*^(*H*)^(*t*) − *B*^*H*^(*τ*) does not depend on the starting time *t* but just on the time lag *t* − *τ*.

The GLE is an extension of the Langevin equation to account for memory effects in the particle-environment interactions. To do that, one generalizes the Stokes drag term to a convolution of the velocity of the particle with a memory kernel representing a generalized time-dependent friction (36). The random fluctuating forces, describing the collisions between the particles composing the environment and the Brownian particle, do not produce a white noise (as in the Langevin dynamics) but a colored noise related to the generalized time-dependent friction through the fluctuation-dissipation theorem. Of course, crowding effects may render the Gaussianity of viscoelastic systems invalid (see, for instance, (12) for membrane systems and (18) for three-dimensional systems).

Another important class is the one of the continuous-time random walks (CTRWs) (22). Differently from the FBM-GLE family, in which motion is fueled by Gaussian noise, CTRWs naturally have non-Gaussian probability densities. Within the CTRW description, particles undergo a series of displacements in a homogeneous medium according with a waiting-time distribution. Anomalous transport is connected with a power-law tail in the waiting-time distribution such that even the mean waiting time is infinite. The central-limit theorem does not apply, as longer and longer waiting times are sampled.

Finally, another important category is represented by the Lorentz models (22), which consider spatially disordered environments in which the particle explores fractal-like structures, leading to anomalous dynamics.

Recently, in the framework of the GLE model, hard exponential and soft power-law truncation (tempering) of power-law memory kernels (37), as well as exponentially truncated three-parameter Mittag-Leffler-based memory kernels (38), have been used to quantitatively reproduce the transition from subdiffusive to normal diffusion. Both exponential and soft power-law truncation imply the introduction of a characteristic crossover time, related to a maximal correlation time in the driving noise. Applications of these models to the analysis of the MSD of lipids in simple lipid bilayers have shown that the crossover time is related to the timescale of mutual exchange between lipids (32,39). Compared to simply combining an anomalous and a normal diffusion law for the MSD, such phenomenological models naturally yield the emergence of subdiffusive-to-normal crossover dynamics into a unique model, in which the type of truncation governs the crossover shape.

To address this problem in the case of laterally diffusing membrane proteins, here we propose a GLE-based model in which the memory kernel is borrowed from a viscoelastic representation of the lipid membrane (22,40, 41, 42, 43, 44, 45). Specifically, we represent the kernel in terms of a viscous (instantaneous) and an elastic (noninstantaneous) term modeled through, respectively, a Dirac *δ* function and the solution of the Prabhakar fractional derivative (46,47), i.e., a three-parameter Mittag-Leffler-based function (48,49). We note that the three-parameter Mittag-Leffler function has also been used in different contexts to model viscoelastic effects (50, 51, 52). We show that imposing a specific relationship between the parameters of the three-parameter Mittag-Leffler function naturally yields the emergence of the different dynamical regimes of the protein (ballistic, subdiffusive, and Brownian) and the crossover between them, with a well-defined finite diffusion coefficient, without introducing hard exponential truncation function (used in the mathematical study of (38)). The spectrum of relaxation times underlying the transition from the subdiffusive to the Brownian regime is derived. The reliability of the proposed GLE model is tested versus the MSD data of a protein (the muscarinic M2 receptor) diffusing in a mixed membrane (containing some of the most abundant species in neuronal cell) and in simpler bilayers made of pure POPC (1-palmitoyl-2-oleoyl-sn-glycero-3-phosphocholine) and POPC/cholesterol 50:50. MSD data are produced by molecular dynamics (MD) simulations. Although not exhaustive, this analysis supports the reliability of the proposed model in providing a consistent picture of protein diffusion. The results on mixed membrane are presented in the main text, and the ones on simpler bilayers are presented as Supporting materials and methods, Section IV. The results are then discussed all together in the main text.

From a continuum perspective, the reliability of the proposed kernel in describing the transition among the different dynamical regimes suggests this function as a possible candidate to describe the time-dependent membrane response within the constitutive equation (as done in (53) for a simpler model). This topic will be addressed in a forthcoming work together with a systematic study on the dependence of the Mittag-Leffler parameters on the membrane composition.

## Materials and methods

### Generalized Langevin equation for protein diffusion

Our aim is to model the transition of a protein diffusing laterally in a lipid bilayer from ballistic to subdiffusive to Brownian motion in terms of a GLE of the form

(1) $$\begin{array}{ll} M\frac{dU_{c}}{dt}\left(t\right) & =-\int_{0}^{t}\zeta \left(t-u\right)U_{c}\left(u\right)\;du+\Xi \left(t\right), \end{array}$$

where ***U***_*c*_(*t*) ∈ $R^{2}$ denotes the protein’s center-of-mass velocity in the *xy* plane, *M* is the protein mass, *ζ* is the generalized friction, and **Ξ**(*t*) = {Ξ^*i*^(*t*)}_*i* = *x*,*y*_ is a two-dimensional Gaussian distributed colored noise that satisfies the fluctuation-dissipation theorem

(2) $$\left\langle {\text{Ξ}}^{i}\left(t\right)\;{\text{Ξ}}^{j}\left(u\right)\right\rangle =2\;k_{B}T\;\zeta \left(t-u\right)\delta_{ij},\;i=x,y.$$

Here, we model the friction term by

(3) $$\zeta \left(t\right):=M\left(\omega_{s}^{2}\delta \left(\frac{t}{\tau_{s}}\right)+\omega_{0}^{2}{\left(\frac{t}{\tau }\right)}^{\nu -1}E_{\lambda ,\nu }^{\delta }\left[-{\left(\frac{t}{\tau }\right)}^{\lambda }\right]\right),$$

where *ω*_*s*_ = 1/*τ*_*s*_ and *ω*_0_ have the physical dimension of a frequency and $E_{\lambda ,\nu }^{\delta }$ denotes the three-parameter Mittag-Leffler function, also called the Prabhakar function (48,49), with the additional parameter *τ* setting the observation timescale of the noninstantaneous component. The function $e_{\lambda ,\nu }^{\delta }\left(t/\tau \right):={\left(t/\tau \right)}^{\nu -1}E_{\lambda ,\nu }^{\delta }$[−*t*/*τ*)^*λ*^] is sometime referred to as the generalized three-parameter Mittag-Leffler function.

The time-dependent functions are dimensionless, and the generalized friction *ζ* has the physical dimension of [*Mt*^−2^]. The normalized function *ζ*/*M* is generally referred to as the memory function. For physical reasons, to have a monotonic kernel function *ζ*, which ensures a monotonic energy decay in isolated systems and a non-negative spectral distribution, the parameters of the Mittag-Leffler function have to satisfy the relation (54) 0 < *λ* ≤ 1 and 0 < *λδ* ≤ *ν* ≤ 1. We refer to Appendix A for an illustration of the asymptotic behavior of the three-parameter Mittag-Leffler function. A survey on the three-parameter Mittag-Leffler function and the generalized three-parameter Mittag-Leffler function, with varying the parameters *λ*, *ν*, and *δ* in the intervals to ensure complete monotonicity, is presented in the Supporting materials and methods, Section II. The numerical implementations adopted in this work to evaluate the Mittag-Leffler functions are illustrated as well (see Supporting materials and methods, Section I).

Note that by setting $e_{\lambda ,\nu }^{\delta }$(*t*/*τ*) equal to 1 in Eq. 3, the model reduces to the confined diffusion in a harmonic potential, where *ω*_0_ is the characteristic frequency of the confining potential and the corresponding MSD tends to the plateau value 2*dk*_*B*_*T*/(*M*$\omega_{0}^{2}$) for *t* → ∞ (*d* being the dimension of the problem).

Instead, replacing $e_{\lambda ,\nu }^{\delta }$(*t*/*τ*) with a simple exponential function exp(−*t*/*τ*) leads to the diffusion in a transient confining harmonic potential with escape time *τ*, which represents the transition from a high-frequency elastic regime to a low-frequency viscous regime (53). The corresponding MSD shows a transient plateau, and then, for time lags larger than *τ*, the standard Brownian dynamics is recovered with diffusion coefficient *k*_*B*_*T*/$\left[M\left(\omega_{s}+\omega_{0}^{2}\tau \right)\right]$.

$e_{\lambda ,\nu }^{\delta }$(*t*/*τ*) being a generalization of the exponential function, which can be written as a superposition of exponential decay rates *f* (54,55),

(4) $$e_{\lambda ,\nu }^{\delta }\left(\frac{t}{\tau }\right)={\left(\frac{t}{\tau }\right)}^{\nu -1}E_{\lambda ,\nu }^{\delta }\left[-{\left(\frac{t}{\tau }\right)}^{\lambda }\right]=\int_{0}^{\infty }p_{\lambda \nu }^{\delta ,\tau }\left(f\right)e^{-ft}df,$$

where

(5) $$\begin{array}{l} p_{\lambda \nu }^{\delta ,\tau }\left(f\right)=\frac{\tau {\left(f\tau \right)}^{\lambda \delta -\nu }\text{sin}\left[\pi \left(\nu -\lambda \delta \right)+\delta \theta_{\lambda }^{\tau }\left(f\right)\right]}{\pi {\left[{\left(f\tau \right)}^{2\lambda }+2{\left(f\tau \right)}^{\lambda }\text{cos}\left(\pi \lambda \right)+1\right]}^{\delta /2}},\\ \theta_{\lambda }^{\tau }\left(f\right)=\text{arg}\left[{\left(-f\tau \right)}^{\lambda }+1\right], \end{array}$$

for 0 < *λ* ≤ 1 and 0 < *λδ* ≤ *ν* ≤ 1, one can see the proposed model as a generalization of the diffusion in a transient confining potential of frequency *ω*_0_ with a relaxation rate spectrum given by $p_{\lambda \nu }^{\delta ,\tau }$(*f*). The parameters *λ*, *ν*, *δ*, and *τ* shape the form and the observation timescale of $p_{\lambda \nu }^{\delta ,\tau }$(*f*). A survey of the impact of these parameters on $p_{\lambda \nu }^{\delta ,\tau }$(*f*) is given in the Supporting materials and methods, Section II.

### Solution of the model and statistical observables

To solve the GLE model Eq. 1, we adopt standard methods for stochastic differential equations as used in (49). Applying the Laplace transform to Eq. 1 and denoting the Laplace transformed functions with the superscript ˆ, it reduces to

(6) $$M\;\left(s\;{\hat{U}}_{c}\left(s\right)-U_{c}\left(0\right)\right)=-\hat{\zeta }\left(s\right)\;{\hat{U}}_{c}\left(s\right)+\hat{\Xi }\left(s\right),$$

and solving for the velocity vector ***Û***_*c*_, we obtain

(7) $${\hat{U}}_{c}\left(s\right)=M\;U_{c}\left(0\right)\;\hat{g}\left(s\right)+\hat{\Xi }\left(s\right)\;\hat{g}\left(s\right),$$

where we have defined the so-called $\hat{g}$(*s*) relaxation function given by

(8) $$\hat{g}\left(s\right):=\frac{1}{Ms+\hat{\zeta }\left(s\right)}.$$

The Laplace transform of *ζ* reads (49)

(9) $$\hat{\zeta }\left(s\right)=\xi_{s}+\xi_{p}\frac{{\left(\tau s\right)}^{\delta \lambda -\nu }}{{\left(1+{\left(\tau s\right)}^{\lambda }\right)}^{\delta }},$$

where we have introduced, for simplicity,

(10) $$\begin{array}{l} \xi_{s}:=M\omega_{s},\\ \xi_{p}:=M\omega_{p},\\ \omega_{p}:=\omega_{0}^{2}\tau . \end{array}$$

The parameters *ξ*_*s*_ and *ξ*_*p*_ have the dimension of a friction, and *ω*_*p*_ has the dimension of a frequency.

Now, by applying the inverse Laplace transform to Eq. 7, one obtains the formal solution for the velocity vector by

(11) $$\begin{array}{l} U_{c}\left(t\right)=\left\langle U_{c}\left(t\right)\right\rangle +\int_{0}^{t}g\left(t-u\right)\;\Xi \left(u\right)\;du,\\ \left\langle U_{c}\left(t\right)\right\rangle =M\;U_{c}\left(0\right)\;g\left(t\right). \end{array}$$

By taking the scalar product of Eq. 11 with ***U***_*c*_(0) and then taking the average, one obtains the velocity autocorrelation function (49,56, 57, 58, 59):

(12) $$C_{v}\left(t\right):=\left\langle U_{c}\left(t\right)\cdot U_{c}\left(0\right)\right\rangle =2k_{B}T\;g\left(t\right),$$

where we used $\left\langle U_{c}\left(0\right)\cdot \Xi \left(u\right)\right\rangle$ = 0 and $\left\langle U_{c}\left(0\right)\cdot U_{c}\left(0\right)\right\rangle$ = 2*k*_*B*_*T*/*M*. Knowing the velocity autocorrelation function (VACF), one can derive the MSD from the well-known convolution relation (60)

(13) $$\left\langle \text{Δ}X^{2}\left(t\right)\right\rangle :=\left\langle \left\|X_{c}\left(t\right)-X_{c}{\left(0\right)}^{2}\right\|\right\rangle =2d\int_{0}^{t}\left(t-\tau \right)C_{v}\left(\tau \right)\;d\tau ,$$

where ***X***_*c*_ is the center-of-mass position vector related to the velocity vector through the time derivative ${\dot{U}}_{c}$ = ***X***_*c*_ and *d* is the dimension of the physical space (we will set *d* = 2 for future applications).

In the Laplace domain, Eq. 13 reads

(14) $$\left\langle \text{Δ}{\hat{X}}^{2}\left(s\right)\right\rangle =2dk_{B}T\frac{\hat{g}\left(s\right)}{s^{2}}=2dk_{B}T\;\hat{I}\left(s\right),$$

where we have introduced the second relaxation function *Î*(*s*) defined by

(15) $$\hat{I}\left(s\right):=\frac{\hat{g}\left(s\right)}{s^{2}}=\frac{1}{Ms^{3}+s^{2}\;\hat{\zeta }\left(s\right)}.$$

Finally, the time-dependent diffusion coefficient can be defined as (9,59)

(16) $$D\left(t\right):=\frac{1}{2d}\frac{d\left\langle \text{Δ}X_{c}^{2}\right\rangle }{dt}\left(t\right),$$

whose physical dimension is [*L*^2^/*t*]. In the limit *t* → ∞, this simply reduces to the standard definition of the Brownian diffusion coefficient *D*_∞_, and the ratio *D*(*t*)/*D*_∞_ accounts for the deviation over time from the Brownian diffusion. Then, the time derivative of Eq. 13 yields

(17) $$D\left(t\right)=K_{B}T\;H\left(t\right),$$

where we have introduced the third relaxation function

(18) $$H\left(t\right):=\int_{0}^{t}g\left(\tau \right)\;d\tau .$$

We note that the Laplace transform of the diffusion coefficient is

(19) $$D\left(s\right)=k_{B}T\frac{\hat{g}\left(s\right)}{s},$$

and therefore,

(20) $$\hat{H}\left(s\right)=k_{B}T\;\frac{\hat{g}\left(s\right)}{s}.$$

We have reduced the problem of studying the protein diffusion throughout the lipid membrane to that of computing the inverse Laplace transform of the relaxation functions Eqs. 8, 15, and 20 so that the correlation functions of interest are found:

(21) $$\begin{array}{l} C_{v}\left(t\right)=2k_{B}T\;g\left(t\right),\\ D\left(t\right)=k_{B}T\;H\left(t\right),\\ \left\langle \text{Δ}X^{2}\left(t\right)\right\rangle =4\;k_{B}T\;I\left(t\right). \end{array}$$

One could be tempted to compute analytically the inverse Laplace transform of the relaxation functions $\hat{g}$, $\hat{H}$, and *Î*. Unfortunately, this is not always possible, and it depends on the mathematical expression of the kernel function *ζ*. Nevertheless, one can exploit numerical methods, as we will see later, to obtain numerical solutions for the observables expressed in Eq. 21.

### Asymptotic behaviors of statistical observables

The short-time behavior of the kernel function *ζ*(*t*) defined by Eq. 3 can be obtained by taking the first term in the series representation of the Mittag-Leffler function (Eq. 35 in Appendix A), which is

(22) $$E_{\lambda ,\nu }^{\delta }\left(-z\right)\approx \frac{1}{\text{Γ}\left(\nu \right)},\;\left(\left|z\right|\ll 1\right),$$

and we obtain the short-time kernel function *ζ*_*S*_ by

(23) $$\zeta_{S}\left(t\right)=\xi_{s}\;\delta \left(t\right)+\frac{\xi_{p}}{\tau^{\nu }}\frac{t^{\nu -1}}{\text{Γ}\left(\nu \right)},\;\left(t\ll \tau \right),$$

whose Laplace transform reads

(24) $${\hat{\zeta }}_{S}\left(s\right)=\xi_{s}+\frac{\xi_{p}}{\tau^{\nu }}s^{-\nu }.$$

By substituting the asymptotic kernel function in the relaxation functions, we get the short-time asymptotic relaxation functions

(25) $$\begin{array}{l} {\hat{g}}_{S}\left(s\right):=\frac{1}{Ms+\xi_{s}+\frac{\xi_{p}}{\tau^{\nu }}s^{-\nu }},\\ {\hat{H}}_{S}\left(s\right):=\frac{{\hat{g}}_{S}\left(s\right)}{s}=\frac{1}{Ms^{2}+\xi_{s}s+\frac{\xi_{p}}{\tau^{\nu }}s^{-\nu +1}},\\ {\hat{I}}_{S}\left(s\right):=\frac{{\hat{g}}_{S}\left(s\right)}{s^{2}}=\frac{1}{Ms^{3}+\xi_{s}s^{2}+\frac{\xi_{p}}{\tau^{\nu }}s^{-\nu +2}}. \end{array}$$

By adopting the methods introduced in (61,62), we can apply the inverse Laplace transform so as to obtain the analytic behavior of the statistical observables at short time in accordance with definitions Eq. 21, namely

(26) $$\begin{array}{l} C_{v}^{S}\left(t\right)\approx \frac{2\;k_{B}T}{M}\left[1-\omega_{s}t\right],\\ D^{S}\left(t\right)\approx \frac{k_{B}T}{M}\left[1-\frac{\omega_{s}t}{2}\right]\;t,\\ \langle \text{Δ}X^{2}\rangle^{S}\left(t\right)\approx \frac{2\;k_{B}T}{M}\left[1-\frac{\omega_{s}t}{3}\right]\;t^{2}. \end{array}$$

Here, one can see that *ω*_*s*_ defines the timescale of the end of the ballistic regime.

The long-time behavior can be obtained by imposing the asymptotic regime $\frac{M}{\xi_{s}}s$ ≪ 1 in the relaxation functions, which, applied to Eqs. 8, 20, and 15, respectively, yields the asymptotic relaxation functions

(27) $$\begin{array}{l} {\hat{g}}_{B}\left(s\right):=\frac{1}{\xi_{s}}\left(\frac{1}{1+\frac{\varphi }{\tau^{\nu }}\frac{s^{\delta \lambda -\nu }}{{\left(\tau^{-\lambda }+s^{\lambda }\right)}^{\delta }}}\right),\\ {\hat{H}}_{B}\left(s\right):=\frac{1}{\xi_{s}}\left(\frac{1}{s+\frac{\varphi }{\tau^{\nu }}\frac{s^{\delta \lambda -\nu +1}}{{\left(\tau^{-\lambda }+s^{\lambda }\right)}^{\delta }}}\right),\\ {\hat{I}}_{B}\left(s\right):=\frac{1}{\xi_{s}}\left(\frac{1}{s^{2}+\frac{\varphi }{\tau^{\nu }}\frac{s^{\delta \lambda -\nu +2}}{{\left(\tau^{-\lambda }+s^{\lambda }\right)}^{\delta }}}\right), \end{array}$$

where we introduced the dimensionless parameter *φ* = *ξ*_*p*_/*ξ*_*s*_. As for the short-time behavior, we adopt the same methods introduced in (61,62) so that the inverse Laplace transforms of the above relaxation functions lead to the analytical expression for the statistical observables.

(28) $$\begin{array}{l} C_{v}^{B}\left(t\right)\approx 2\;k_{B}T\frac{\tau^{-\delta \lambda +\nu }}{\xi_{p}}\frac{t^{\delta \lambda -\nu -1}}{\text{Γ}\left(\delta \lambda -\nu \right)}-2\;k_{B}T\frac{\delta \tau^{\lambda }}{\xi_{s}}t^{-\lambda -1}\overset{\infty }{\underset{n=0}{\sum }}{\left(-\frac{\varphi }{\tau^{\nu -\delta \lambda }}\right)}^{n}\frac{n\;t^{\left(\nu -\delta \lambda \right)n}}{\text{Γ}\left[\left(\nu -\lambda \delta \right)n-\lambda \right]},\\ D^{B}\left(t\right)\approx k_{B}T\frac{\tau^{-\delta \lambda +\nu }}{\xi_{p}}\frac{t^{\delta \lambda -\nu }}{\text{Γ}\left(\left(\delta \lambda -\nu \right)+1\right)},\\ \langle \text{Δ}X^{2}\rangle^{B}\left(t\right)\approx 4\;k_{B}T\frac{\tau^{-\delta \lambda +\nu }}{\xi_{p}}\frac{t^{\delta \lambda -\nu +1}}{\text{Γ}\left(\left(\delta \lambda -\nu \right)+2\right)}. \end{array}$$

To recover the Brownian regime in the long-time limit, *D*(*t*) has to approach a constant *D*_∞_ > 0. From the second equation in Eq. 28, one notices that this requirement is satisfied if and only if

(29) δλ−ν=0.

The exact mathematical expression for *D*_∞_ can be obtained considering the nonapproximated expression for $\hat{H}$(*s*), defined by Eq. 20, which under the constraint Eq. 29 reads

$$\hat{D}\left(s\right)=\frac{k_{B}T}{s}\left(\frac{1}{M\;s+\xi_{s}+\frac{\xi_{p}}{{\left(1+{\left(\tau \;s\right)}^{\lambda }\right)}^{\delta }}}\right).$$

By taking the limit *s* → ∞, the leading term is

$$\hat{D}\left(s\right)\approx \frac{k_{B}T}{s}\frac{1}{\xi_{s}+\xi_{p}}.$$

The inverse Laplace transform provides then the exact expression for the asymptotic diffusion coefficient

(30) $$D_{\infty }=\frac{k_{B}T}{\xi_{s}+\xi_{p}}.$$

By imposing the constraint Eq. 29 in expressions Eq. 28, we finally get the asymptotic behavior of the VACF:

(31) $$C_{v}^{B}\left(t\right)\approx 2\;k_{B}T\delta \tau^{\lambda }\frac{t^{-\lambda -1}}{\text{Γ}\left(-\lambda \right)}\frac{\xi_{p}/\xi_{s}^{2}}{{\left(1+\xi_{p}/\xi_{s}\right)}^{2}}=\frac{2\;k_{B}T}{M}\delta \tau^{\lambda }\frac{t^{-\lambda -1}}{\text{Γ}\left(-\lambda \right)}\frac{\omega_{p}/\omega_{s}^{2}}{{\left(1+\omega_{p}/\omega_{s}\right)}^{2}},$$

where we impose λ∉Z and, by definition, 1/Γ(0) = 0. We note that in the limit of *ξ*_*p*_ ≫ *ξ*_*s*_, we have

(32) $$C_{v}^{B}\left(t\right)\approx \frac{2\;k_{B}T}{M}\frac{\delta \tau^{-\lambda }}{\xi_{p}}\frac{t^{-\lambda -1}}{\text{Γ}\left(-\lambda \right)}.$$

Similarly, for the MSD, we have

(33) $$\left\langle \text{Δ}X{\left(t\right)}^{2}\right\rangle \approx \frac{4\;k_{B}T}{\xi_{p}}t,$$

so that the linear Brownian regime in the long time is recovered. Notice that in this limit, the friction is dominated by the component *ξ*_*p*_. Finally, we summarize the physical role of each parameter in Table 1.

**Table 1:** Summary of the physical role of the parameters

Parameters	Physical role
*ω*_*s*_	characteristic frequency setting the timescale *τ*_*s*_ = 1/*ω*_*s*_ of the end of the ballistic regime
*ω*_0_	characteristic frequency of the transient confining potential
*ξ*_*s*_	friction component coming from the instantaneous response
*ξ*_*p*_	friction component coming from the retarded response; it is the leading term in the asymptotic diffusion coefficient
*ξ*_*p*_ + *ξ*_*s*_	total macroscopic friction felt by the protein
τ	timescale of the retarded (elastic) response of the lipid membrane
*δ*, *λ*, *ν*	*δλ* = *ν* to asymptotically get the Brownian regime; they shape the transition from subdiffusive-to-Brownian regime

### Data availability

All the Mathematica codes used to produce the results presented in this manuscript will be made available by the authors upon request.

## Results

We test in this section the developed theoretical model by applying it to a realistic system of a protein in a mixed membrane, as well as performing two control tests of simpler scenarios to verify the coherence and generality of the proposed theoretical model. We proceed by presenting the application first, followed by the control tests.

### M2 muscarinic acetylcholine receptor in mixed membrane

**Figure 1:**
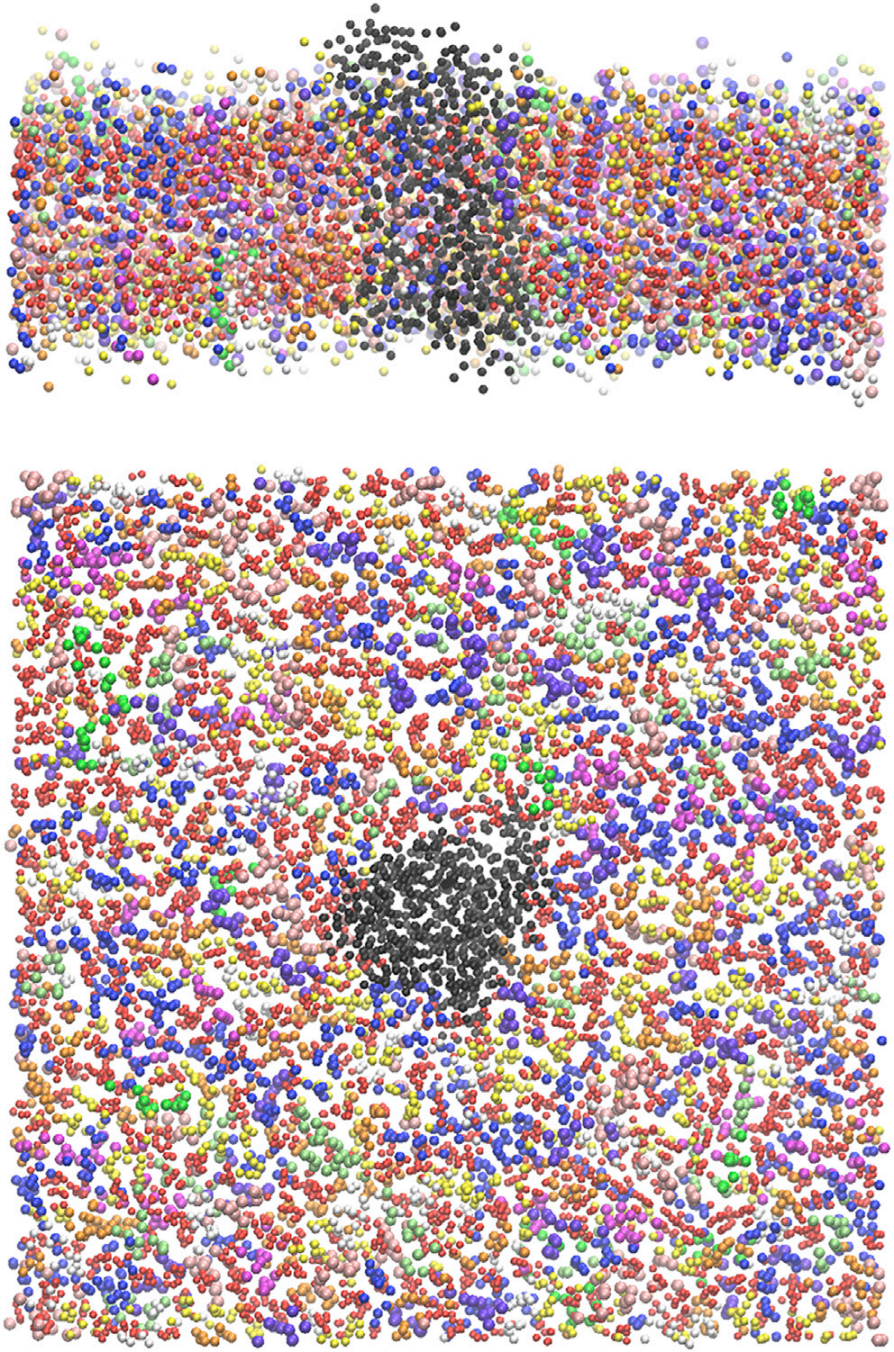
Snapshot of the simulation box created with the Martini force field. For better visualization, we do not illustrate water and ion molecules. We report (*top*) front view and (*bottom*) top view of the membrane-protein system. The protein is represented by the black domain, and the lipids are colored depending on the species. Cholesterol: red, POPC: blue, DOPC (1,2-dioleoyl-sn-glycero-3-phosphocholine): lime (*lighter*) green, PAPI (1-palmitoyl-2-arachidonoyl-sn-glycero-3-phosphoinositol): white, DGPE (1,2-digondoyl-sn-glycero-3-phosphoethanolamine): magenta, DPPC (1,2-dipalmitoyl-sn-glycero-3-phosphocholine): orange, POPS (1-palmitoyl-2-oleoyl-sn-glycero-3-phosphoserine): violet, DPSM (N-palmitoyl-D-sphingomyelin): yellow, PNSM (N-nervonoyl-D-sphingomyelin): pink, POSM (N-oleoyl-D-sphingomyelin): (*darker*) green. To see this figure in color, go online.

As an application, we fitted the model to in silico MSD data of the center of mass of a protein laterally diffusing in a fully hydrated mixed membrane, containing some of the most abundant lipid species in neuronal cell membranes (63). We used as model protein the M2 muscarinic acetylcholine receptor (M2 receptor), an inhibitory class A G-protein-coupled receptor expressed both in the central and parasympathetic nervous systems (64). The inactive form of M2 (64) (Protein Data Bank: 3UON) was embedded in the mixed lipid bilayer (see Appendix B for the lipids composition). The antagonist and the fused T4 lysozyme, which in the original Protein Data Bank structure replaces the third intracellular loop of the receptor, were removed from the system. About 14,000 water molecules were added, as well as Na^+^ and Cl^−^ ions, to neutralize the system and reach a physiological concentration of 0.15 M. A snapshot of the simulated system is shown in Fig. 1. The characteristic size of the system and the simulation details are reported in Appendix B.

#### The in silico MSD and VACF data are extracted from quasiatomistic Martini MD simulations

The theoretical asymptotic behavior of the VACF, as derived from the model by using the MSD best-fit parameters, is compared to in silico VACF data (65, 66, 67). We also compute the in silico MSD of the surrounding lipids. Within this process, we distinguish between NEAR and FAR lipids. NEAR lipids are selected by picking the lipids spending more time within a threshold distance of 3 nm from the protein center of mass (on average, 84 lipids are found within this threshold). Actually, because of the diffusion, lipids initially satisfying the distance criterion may diffuse away and other lipids can enter within the NEAR lipid radius. Hence, we selected the 84 lipids with the largest residence time within the threshold radius. FAR lipids are defined as the complementary ensemble. For each group of lipids, the average representative MSD (VACF) is calculated as the mass weighted sum of the MSD (VACF) of the lipids in the group.

#### Gaussianity check

The protein dynamics in a lipid membrane has been observed to be non-Gaussian in several cases such as, for instance, compartmentalization and crowding (12). In our case, we work at infinite protein dilution, but the membrane is mixed and contains a cholesterol concentration of ∼50%, which could cause local inhomogeneities.

**Figure 2:**
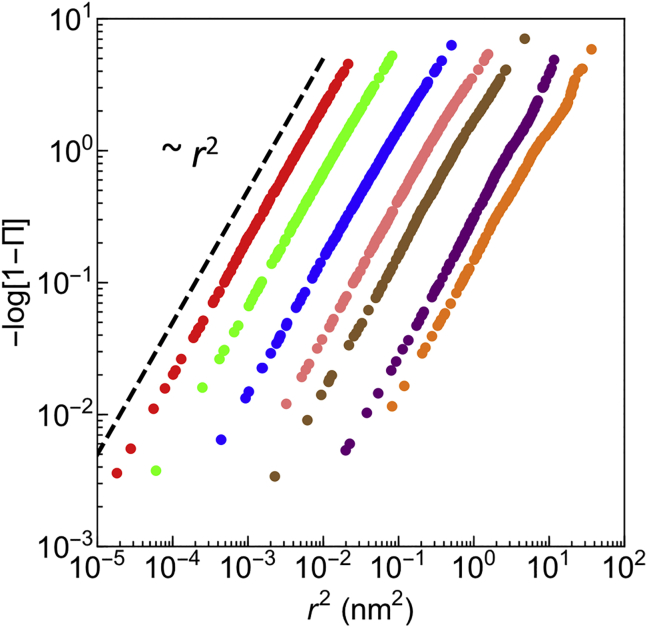
Cumulative distribution Π(*r*^2^, Δ) with Δ = 1, 10, 100, 400, 1000, 5000, and 10,000 ns (from *left* to *right*). The dashed line corresponds to a theoretical curve proportional to *r*^2^. To see this figure in color, go online.

As a first step, we thus verified whether sizable deviations from a Gaussian process are observed. By adopting the same approach as in (12), we have computed the cumulative distribution Π(*r*^2^, Δ) for the squared displacements of the protein varying the time lag Δ as shown in Fig. 2. Here, we recall that the cumulative distribution of the square displacements for the two-dimensional motion is Π(*r*^2^, Δ) = $\int_{0}^{r}$*P*(**r**′, Δ)2*πr*′*dr*′, where *P*(**r**, Δ) is the propagator (6,68,69). In the case of a Gaussian (anomalous) diffusion, it takes the form *P*(**r**, Δ) = exp[−*r*^2^/2*σ*_Δ_)]/(2*πσ*_Δ_), with *σ*_Δ_ = 2*D*_*α*_Δ^*α*^, which yields the cumulative distribution Π(*r*^2^, Δ) = 1 − exp[−*r*^2^/(4*D*_*α*_Δ^*α*^)]. The plot of −log[1 − Π(*r*^2^, Δ)] vs. *r*^2^ in Fig. 2 displays a reasonable power-law scaling with exponent 2. Thus, despite the high cholesterol content of the model bilayer (∼50%), the protein dynamics turns out to be Gaussian and thus compatible with the model hypothesis.

Notice that the model has not been fitted to the MSD of the mixed membrane lipids because, as observed by us in this work (see Supporting materials and methods, Section V) as well as by other authors dealing with membranes with cholesterol concentrations of the order of ∼50% (70), lipids dynamics deviates from a truly Gaussian process. However, we derived the dynamical properties of the embedding bilayer from a phenomenological (model-free) analysis of lipids’ MSD and VACF.

#### Fitting the model to MD data

After this test, the model was then tested against the lateral MSD data of the protein extracted from the MD simulations. Two different flavors of the model have been implemented, corresponding to different choices of the free parameters (***θ***) of the model.

1 Model M1: ***θ*** = (*ω*_*s*_, *ω*_*p*_, *τ*, *λ*, *δ*), with constraint *ν* = *δλ*; and
2 Model M2: ***θ*** = (*ω*_*s*_, *ω*_*p*_, *τ*, *λ*), with constraint *δ* = 1 together with the condition *δλ* = *ν*, then gives *λ* = *ν.*

Model M1 corresponds to a three-parameter Mittag-Leffler function and is the more generic model, whereas model M2 reduces to a two-parameter Mittag-Leffler function, which has already been used in the literature in the context of viscoelasticity (71). The mass of the protein and the temperature are fixed to *M* = 41,697 g/mol and *T* = 310 K in both implementations. The expected values of the parameters ***θ*** and their uncertainties have been evaluated by using a Bayesian approach with a flat (uninformative) prior distribution for the parameters (72), i.e., all the parameter values are assumed to be equally probable before analyzing the data. Details on the data analysis are presented in Appendix C.

Because of the mathematical complexity of the MSD model, we do not provide an analytical expression for the MSD in the time domain. Hence, to carry out the fit, we exploit its simple analytical expression in the Laplace space. In fact, combining Eqs. 9, 20, and 21, under the condition *δλ* = *ν*, the model is written (in the Laplace domain)

(34) $$\left\langle {\hat{\text{Δ}X}}^{2}\right\rangle \left(s\right)=\frac{4\;k_{B}T}{M}\frac{1}{s^{2}}\frac{1}{s+\omega_{s}+\omega_{p}\frac{1}{{\left(1+{\left(\tau s\right)}^{\lambda }\right)}^{\delta }}}.$$

For a given set of parameters, the model is first evaluated in the Laplace space through Eq. 34. Then, this numerical expression is inverse Laplace transformed using the Mathematica function ILT by Horváth et al. (73, 74, 75). This allows one to evaluate the model in the time domain and thus calculate the residuals between the model and MD data for each set of parameters.

Model M1 was investigated by sampling the parameter space over a quite wide grid of values. This analysis shows that the best fit is obtained for *δ* ∼1, which points toward model M2. We thus investigated model M2 with a finer sampling in the parameter space (see Appendix C for the grid of the investigated parameter values). Both models provide a reasonable fit in terms of reduced ${\tilde{\chi }}^{2}$, without significant changes for the best-fit parameters. We thus report here in the main text the data and the analysis relevant to model M2, and the data for model M1 are available in Appendix C.

The best-fit parameters and the 95% confidence intervals are summarized in Table 2 together with the parameters *ω*_0_, *ξ*_*s*_, and *ξ*_*p*_ derived thereof and the Brownian diffusion coefficient *D*_∞_.

**Table 2:** Best-fit parameters, fixed parameters, and parameters derived from the best-fit parameters for model M2

*δ*^a^	*λ*^b^ = *ν*	*τ*^b^ (ns)	*ω*_*s*_^b^ (ps^−1^)	*ω*_*p*_^b^ (ps^−1^)	*ω*_0_^c^ (ps^−1^)	*ξ*_*s*_^c^ (Pa ⋅ s ⋅ *μ*m)	*ξ*_*p*_^c^ (Pa ⋅ s ⋅ *μ*m)	*D*_∞_^c^ (*μ*m^2^ ⋅ s^−1^)
1	$0.715_{0.70}^{0.73}$	$13.4_{12.0}^{15.1}$	$0.98_{0.86}^{1.11}$	$447_{433}^{462}$	$0.183_{0.175}^{0.189}$	$0.067_{0.059}^{0.077}\cdot 10^{-3}$	$0.0309_{0.0299}^{0.0319}$	$0.137_{0.133}^{0.142}$

The sub- and superscripts represent the 95% confidence intervals.

a Fixed parameters.

b Best-fit parameters.

c Parameters derived from the best-fit parameters.

**Figure 3:**
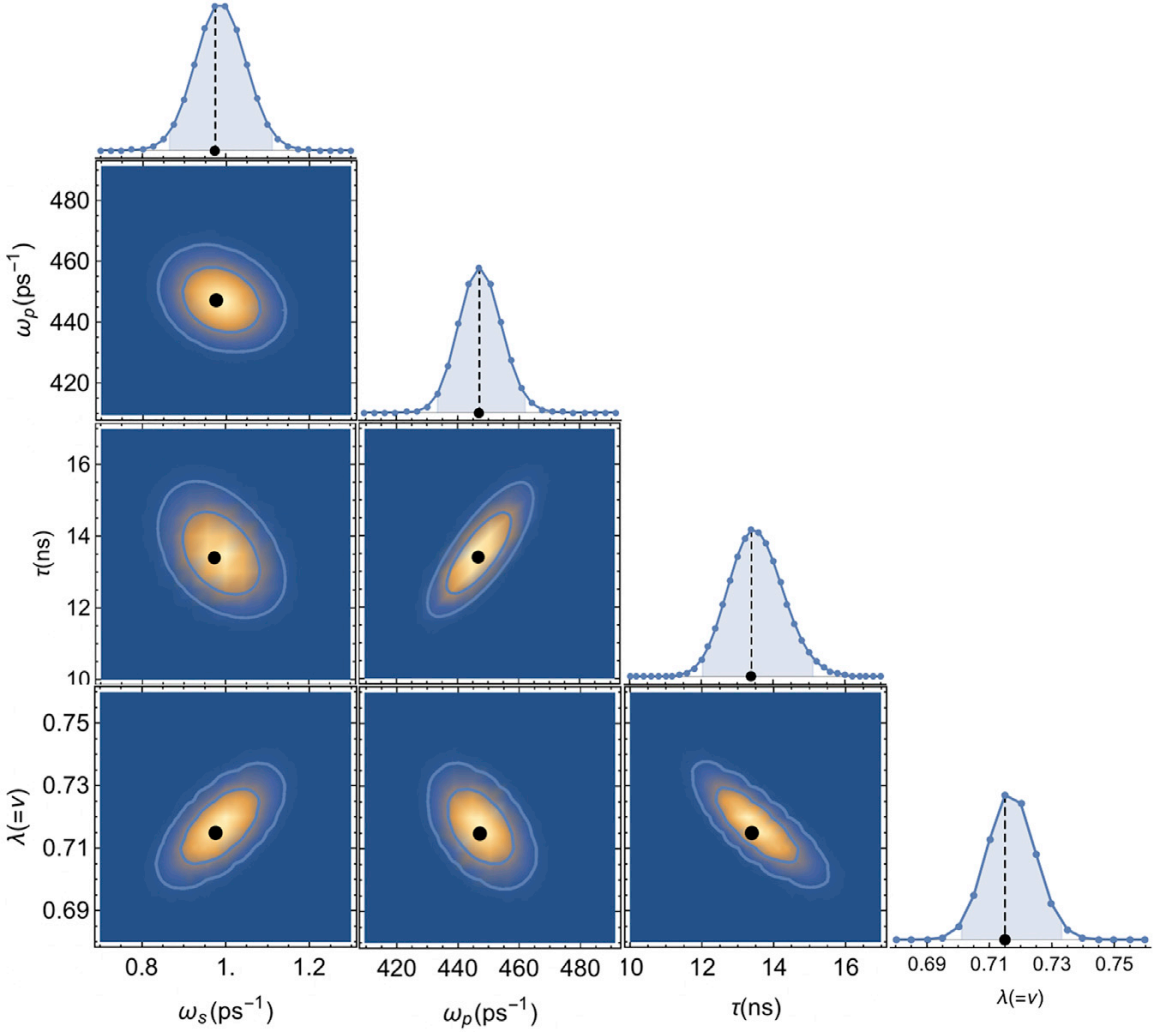
One-dimensional and two-dimensional marginal distributions for model M2. The highlighted area below the one-dimensional marginal distributions corresponds to 95%. The ellipses in the two-dimensional marginal distributions correspond to 68 and 95% confidence regions. The reduced ${\tilde{\chi }}^{2}$ is 0.94. To see this figure in color, go online.

The one-dimensional marginalized posterior distributions, used to estimate the best-fit values and the confidence intervals, are illustrated in Fig. 3 together with the two-dimensional marginalized posterior distributions showing the correlations between the parameters. See Appendix C for more details about their calculations.

**Figure 4:**
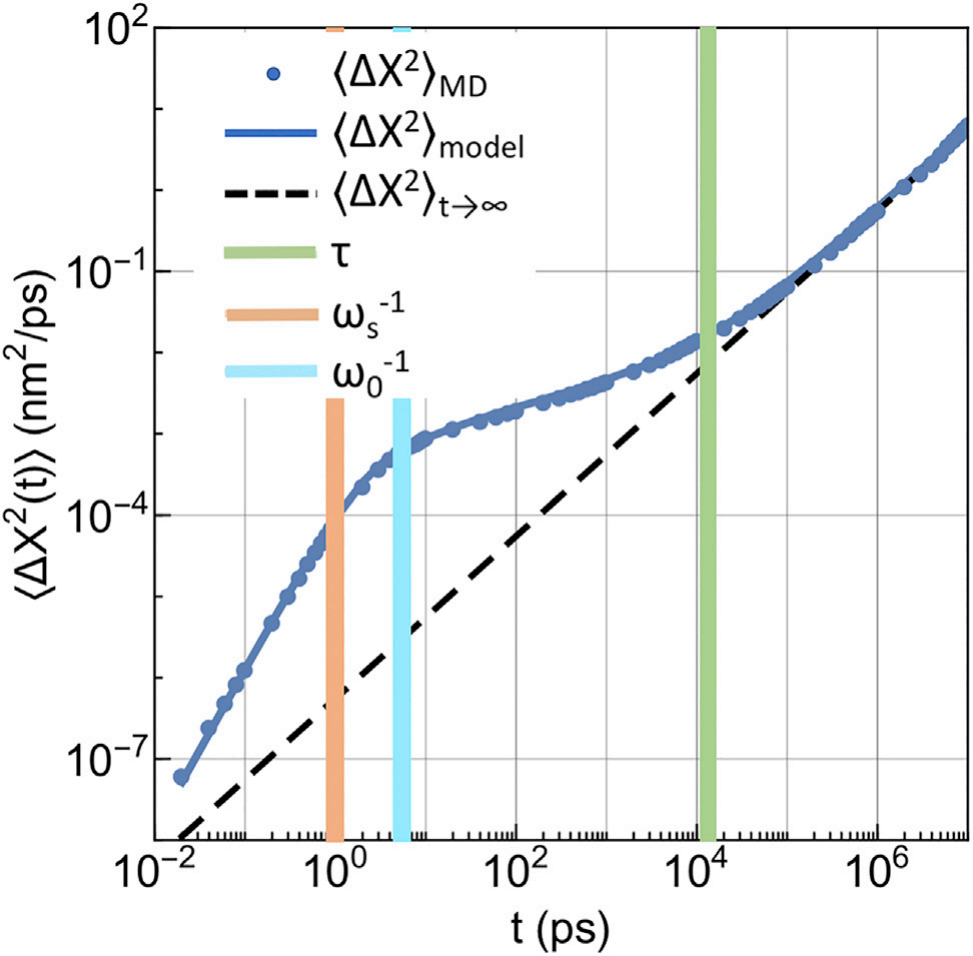
Comparison between the MSD data from MD simulations (*blue dots*) and GLE model (*blue line*). The long-time asymptotic MSD (*black*) and the characteristic times *τ* (*green*) and $\omega_{s}^{-1}$ (*orange*) and $\omega_{0}^{-1}$ (*cyan*) are also shown. To see this figure in color, go online.

The comparison in log-log scale between the model and the numerical MSD data of the protein diffusing in the mixed membrane is shown in Fig. 4. To highlight the physical role of the model parameters, we report in the same figures the asymptotic MSD obtained from the model $\langle \text{Δ}r^{2}\left(t\right)\rangle_{t\to \infty }$ = 4 *D*_∞_ *t*, with *D*_∞_ given by Eq. 30, the characteristic timescale *τ* of the retarded response, the transition timescale 1/*ω*_*s*_ from the ballistic to the subdiffusive regime, and the characteristic timescale 1/*ω*_0_ associated to the frequency of the transient confining potential.

We emphasize that the reported numerical values of the diffusion coefficient *D*_∞_ may suffer from finite size effects because of the periodic boundary conditions and the limited box size used in simulations (29,76). In accordance with the error estimation reported in the literature for systems analogous to the one discussed here (i.e., protein radius ∼1.8 nm, total water thickness 6.2 nm, lateral membrane edge 16.6 nm, Martini force field), an underestimation of ∼40% is expected (29,76).

**Figure 5:**
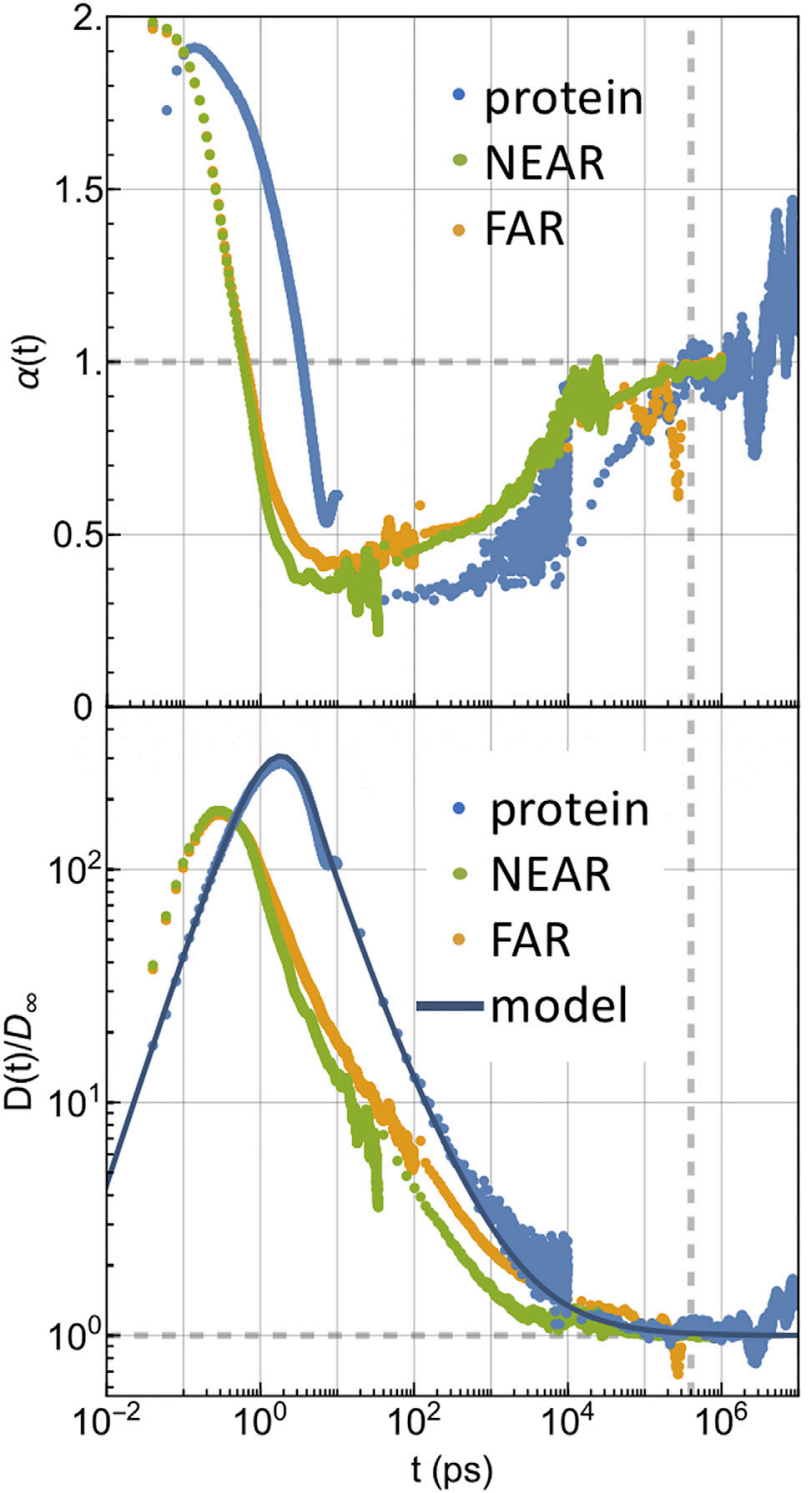
(*Top*) Local MSD dependence on time of protein and NEAR and FAR lipids. *α* = 2 indicates the ballistic regime, *α* = 1 the Brownian regime, and 0 < *α* < 1 the subdiffusive regime. (*Bottom*) Comparison between protein’s time-dependent diffusion coefficient normalized to the value *D*_∞_ = *k*_*B*_*T*/(*ξ*_*s*_ + *ξ*_*p*_) as derived from the model and the numerical counterpart derived from MD simulations. Numerical results for NEAR and FAR lipids as derived from MD simulations are shown as well. Gray lines help to visualize the onset of the Brownian dynamics. To see this figure in color, go online.

The evolution of the protein time-dependent diffusion coefficient, as defined in Eq. 16 in terms of derivative of the MSD, is shown in Fig. 5 upon normalization by *D*_∞_ (*blue curve*). The theoretical values of *D*(*t*) are calculated from the second equation in Eq. 21 by inverse Laplace transforming Eq. 20 through the numerical function by Horváth et al. (73, 74, 75). The values of *D*(*t*) from MD are obtained through the numerical derivative of the MSD. For comparison, the same observables for NEAR and FAR lipids are shown as well (*yellow* and *green curves*). The MSD of NEAR and FAR lipids is shown in the Supporting materials and methods, Section V.

The coefficient *α*(*t*), expressing the phenomenological instantaneous dependence of the MSD on time (MSD ∝ *t*^*α*(*t*)^) as calculated from the logarithmic derivative of the simulated MSD data, is reported in the same figure.

A comparison between protein and NEAR(FAR)-lipids VACFs at short-time lags is given in Fig. 7. Long-time VACF tails are shown in the Supporting materials and methods, Section V.

**Figure 6:**
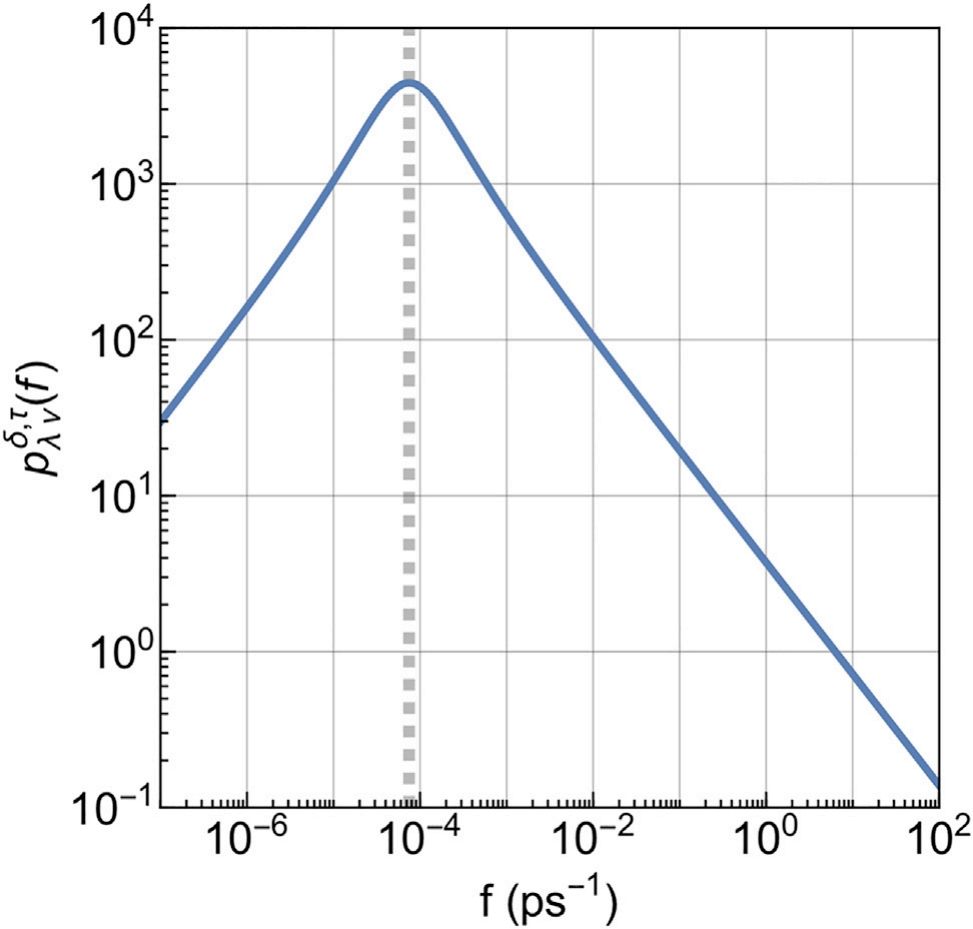
Distribution $p_{\lambda \nu }^{\delta ,\tau }$(*f*) of relaxation rates *f* underlying the memory kernel. The gray line corresponds to 1/*τ*, where *τ* = 13.4 ns. To see this figure in color, go online.

Finally, exploiting the integral representation of $e_{\lambda ,\nu }^{\delta }$(*t*/*τ*) by Mainardi (54,55) (Eq. 4), we plot the relaxation rate spectrum $p_{\lambda \nu }^{\delta ,\tau }$(*f*) (Eq. 5) for the optimally fitted parameters of protein (Fig. 6). This provides the contribution of the different relaxation rates to the memory kernel. 1/*τ* sets the maximum of the spectrum, and *λ* (= *ν*) regulates the width (with the setting *δ* = 1, the spectrum tends to a Dirac *δ* function when *λ* (= *ν*) → 1).

### Liquid argon

To test the ability of Mittag-Leffler functions in capturing small deviations from the ideal Dirac *δ* function kernel predicted for an ideal Brownian system, we analyzed the MSD of liquid argon. The details and results are reported in the Supporting materials and methods, Section III. The memory kernel of argon predicted by the model compares quite well with the numerical calculations reported in the literature (77,78). A comparison between the VACF predicted by the model and the one extracted from MD simulations is shown as well (see Supporting materials and methods).

### Simple membranes with and without protein

As an additional control of the reliability of the model, we apply the workflow used for M2 diffusing in mixed membrane on simpler membrane systems showing a truly Gaussian behavior, such as lipids diffusing in pure POPC, protein (M2) diffusing in pure POPC, and protein (M2) diffusing in POPC/cholesterol 50:50. Note that the Gaussianity of membrane systems in protein-poor conditions with cholesterol concentration up to ∼20% was already established in (12,17,39). The Gaussianity test for the receptor diffusing in pure POPC and in the two-component membrane POPC/cholesterol 50:50 is reported in the Supporting materials and methods, Section IV. The results of the fitting to the MSD data of the three membrane systems are reported in the same section of the Supporting materials and methods, and they are further discussed together with the ones of the protein diffusing in the mixed membrane in the Discussion section below.

## Discussion

Both Figs. 4 and 5 show that the subdiffusive-to-Brownian dynamics transition of the protein lasts from tens to hundreds of nanoseconds and that Brownian dynamics is fully recovered only at time lags of ∼400 ns. This timescale indicates the maximal sizable relaxation time contributing to the memory kernel. Within this timeframe, the protein moves ∼0.5 nm, to be compared with the radius of an average lipid, which is ∼0.4 nm. The local slope *α*(*t*) of the NEAR lipids MSD is ∼0.8–0.9 for time lags of the order of tenths of nanoseconds, but the truly Brownian dynamics (*α* = 1) is fully recovered for both NEAR and FAR lipids at approximately the same timescale of the protein, i.e., 400 ns. Interestingly, this timescale is close to the observed average residence time of the lipids in the NEAR shell (∼350 ns). For larger time lags, NEAR lipids exit from the NEAR shell and start to behave as FAR lipids. Indeed, for *t* → ∞, the MSDs of both NEAR and FAR lipids attain the same limit (hence the same Brownian diffusion coefficient). See Fig. S12 in Supporting materials and methods. Furthermore, by comparing long-time tails of the VACF of both NEAR and FAR lipids to the one of the protein, one observes that negative correlations seem to persist for shorter time lags in the case of NEAR lipids (∼5 ps) compared to FAR lipids and protein (∼15 ps) (see Supporting materials and methods, Section V). In accordance with Eq. 31, long-time anticorrelations of protein’s VACF decay to zero as ∼−*t*^−1 −^ ^*λ*^, where *λ* is 0.71 (see Section V of the Supporting materials and methods for a graphical comparison between the VACF asymptotic behavior predicted by the model and in silico VACF data). Apart from a scaling factor, both NEAR and FAR lipid VACFs show a similar trend. All these facts suggest that protein’s Brownian dynamics recovering is correlated with the lipids’ shell relaxation and that in the subdiffusive regime, protein moves concertedly with its neighbors.

**Figure 7:**
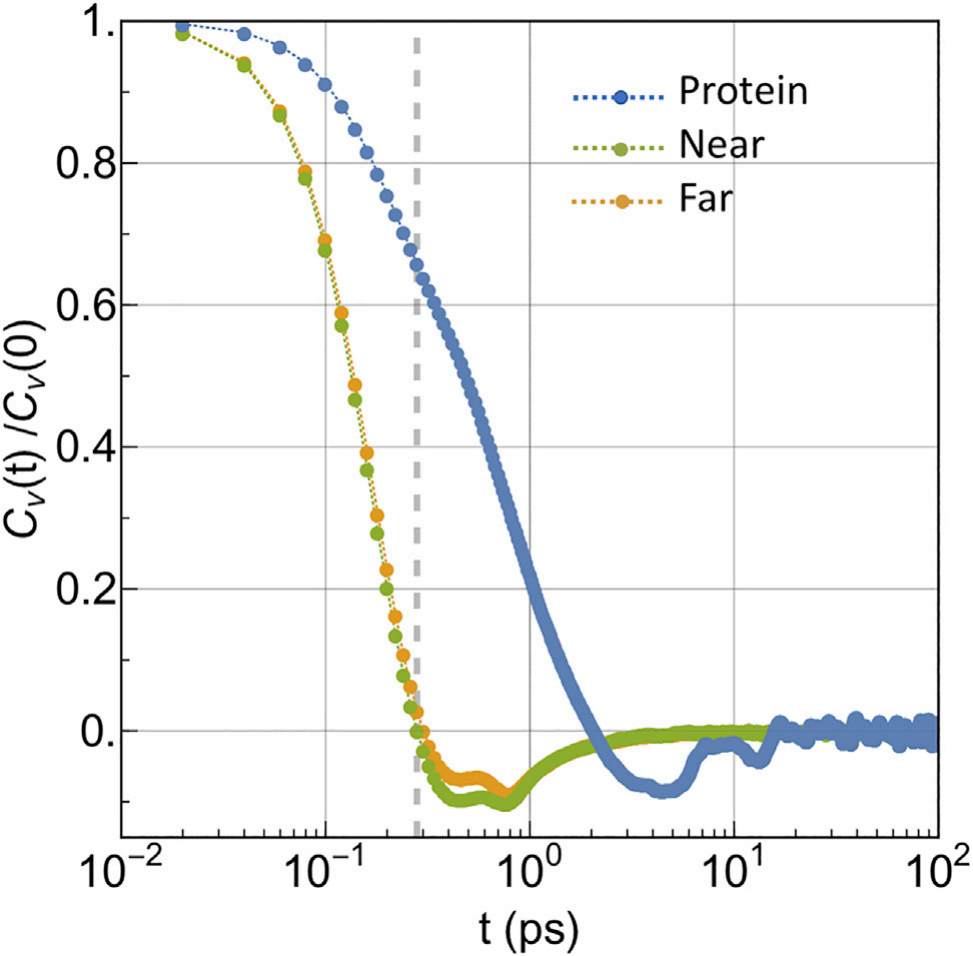
Comparison between the VACF data from MD simulations of protein and NEAR and FAR lipids. Dashed gray line indicates the point at which lipid negative correlations start. To see this figure in color, go online.

Looking at short-time lags, the protein’s VACF shows a shoulder occurring at approximately the timescale at which the lipid’s velocities become anticorrelated (∼0.28 ps for NEAR lipids and 0.3 ps for FAR lipids) (see Fig. 7). This could indicate a first collision event between the protein and the lipid’s shell, after which the protein keeps moving without turning back, whereas lipids turn back. Interestingly, the end of the ballistic regime of the protein occurs at 1/*ω*_*s*_ ≈ 1 ps, which possibly suggests an extended ballistic regime after the first collision event with lipids.

Notice that *ω*_*s*_, *ω*_0_, and *λ* of the M2 protein diffusing in the mixed membrane (cholesterol content ∼58%) are similar to the ones obtained for M2 diffusing in POPC/cholesterol 50:50 (see Table S2), whereas in the case of M2 diffusing in pure POPC, they are substantially smaller (∼40, 80, and 60%, respectively). This suggests that, for a given diffusing system, cholesterol plays a fundamental role in speeding up the ballistic to (sub)diffusive transition, increasing the frequency of the transient confining potential, and decreasing the width of the relaxation rate spectrum. In the presence of cholesterol, protein feels a transient confining potential of higher frequency, but the width of the relaxation rates is narrower. As for the maximum of the relaxation rate spectrum of the protein in the mixed membrane, it occurs at lower frequencies (longer timescales) than for the protein diffusing in POPC/cholesterol 50:50 (1/13.4 ns^−1^ vs. 1/1.8 ns^−1^, respectively). Because the cholesterol content is similar in the two membranes, these preliminary data suggest that the higher heterogeneity of the mixed membrane, compared to the two-component POPC/cholesterol 50:50, is the main factor responsible for this shift of the spectrum to lower frequencies. Interestingly, comparing the value of 1/*τ* of the protein diffusing in pure POPC with the one of the protein diffusing in the two-component POPC/Cholesterol 50:50, one has that cholesterol shifts the relaxation rates spectrum to higher frequencies. All together, these facts suggest that not only does the maximum of the spectrum in the mixed membrane seem to be essentially driven by the heterogeneity of the membrane, but cholesterol alone would possibly act in the opposite direction. A more in-depth analysis is in progress to understand the origin of this slowing down in the mixed membrane.

All these findings suggest that the transient anomalous diffusion effects stem from highly nontrivial interactions of the protein with the fluid made up of lipid molecules behaving as a whole, in contrast to more shapeshifting proteins in water with fluctuating diffusivity (79).

Finally, our preliminary data on pure POPC membrane (see Supporting materials and methods, Section IV and Table S2) indicate that the proposed model is a priori adapted to also describe the dynamics of lipids diffusing in a membrane of pure POPC, although systematic work with varying membrane composition is required to further check the coherence of the parameters.

## Conclusions

The proposed generalized Langevin equation-based model, built on a memory kernel made of an instantaneous viscous component *δ*(*t*) and a retarded (elastic) component $t^{\delta \lambda -1}E_{\lambda ,\delta \lambda }^{\delta }$(−(*t*/*τ*)^*λ*^), proved to be able to describe the transition of the lateral diffusion of a protein from the ballistic to the subdiffusive regime and from the subdiffusive to the Brownian regime when the constraint *ν* = *λδ* is imposed. Notice that the model has been tested and validated at infinite protein dilution for a protein diffusing in a mixed membrane (containing some of the most abundant species in neuronal membranes), as well as in two-component POPC/cholesterol 50:50 and in pure POPC. In all of these conditions, protein dynamics proved to be Gaussian. To the contrary, for lipids diffusing in crowded conditions (high protein concentration or cholesterol concentration higher than 20%), deviations from the Gaussianity hypothesis underlying the proposed model have been reported (12), and it has been shown that the non-Gaussian behavior is related to trapping times distributed according to a power law (80). In our study, we observed sizable deviations from a Gaussian process for the dynamics of the lipids of the mixed membrane, which has a cholesterol content of the order of ∼50%. In all of these conditions, our model cannot be applied.

Our findings suggest that the transient subdiffusive behavior arises from the interactions between the protein and the lipid matrix, inducing a transient confinement with a large distribution of relaxation times. The protein moves in a concerted way with the neighbor lipids. Within the proposed model, the analysis of the selected use cases is compatible with the picture that cholesterol speeds up the ballistic to (sub)diffusive transition, increases the frequency of the transient confining potential, and decreases the width of the relaxation rates spectrum. A systematic study on the effect of different membrane compositions on the distribution of relaxation times is ongoing. A test on pure POPC membrane indicates that the model can also be a priori applied to describe the dynamics of lipids when the Gaussianity conditions are verified (i.e., noncrowded conditions). Finally, we remark that the application of the Mittag-Leffler functions in several relevant stochastic processes, such as the fractional Poisson process and CTRWs, has been discussed in (81). Several applications to model viscoelastic effects can be found in (50, 51, 52,82, 83, 84). Applications of Mittag-Leffler functions in the context of obstructed diffusion (Lorentz gas) and CTRWs are also of potential interest. We cite the work of Hoefling and Franosch (22) on two-dimensional and three-dimensional Lorentz gas with immobile obstacles, which showed ballistic, subdiffusive, and diffusive motion as well as the crossovers between them, and the work of Akimoto et al. (80) on membrane systems in the framework of CTRWs. Mittag-Leffler functions are also applied to describe internal protein dynamics relaxation. To the best of our knowledge, one of the first studies in this context is (85).

In a continuum perspective, one could use this function to model the time-dependent (viscoelastic) membrane response within the constitutive equation.

## Author contributions

V.C. designed the project. L.D.C. and B.S. performed analytical derivation. L.D.C. performed MD simulations. L.D.C. and V.C. performed the analysis. L.D.C., B.S., and V.C. wrote the manuscript.

## Figures and Tables

**Figure 8:**
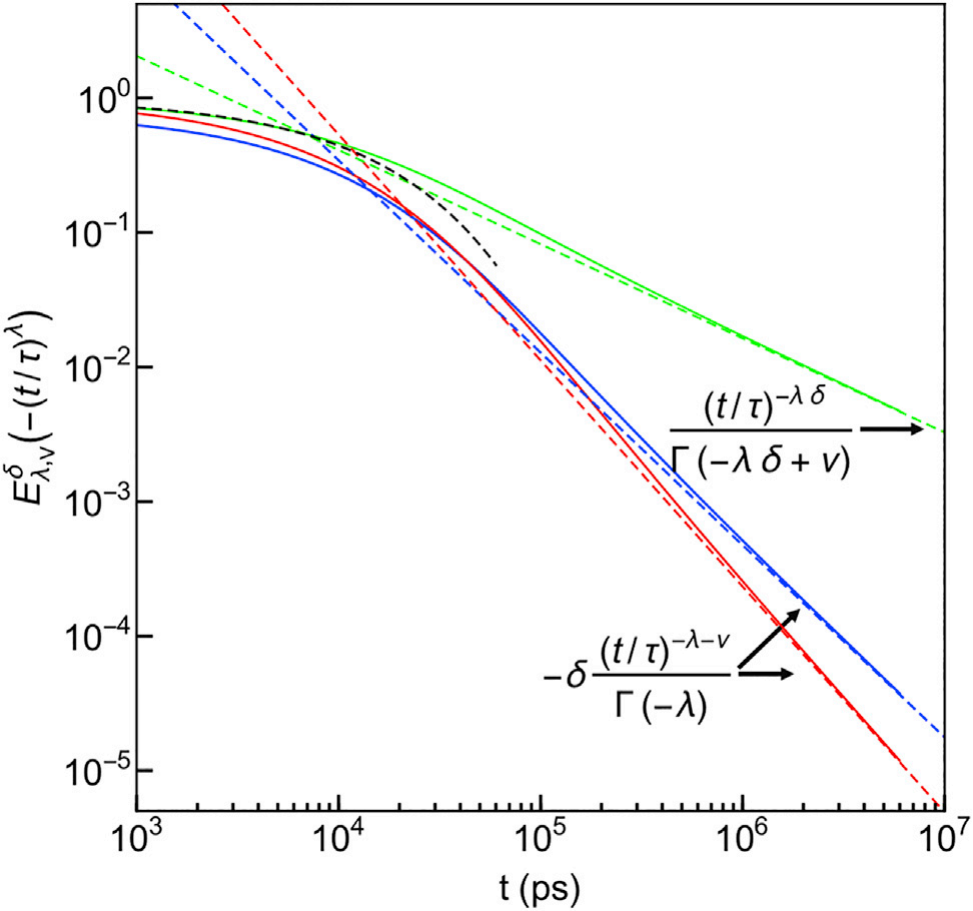
Illustration of the three-parameter Mittag-Leffler function (*solid lines*) for three different set of parameters (*blue*: as presented in Table 2; *red*: *τ* = 13.4 ns, *λ* = 0.7, *ν* = 0.98, *δ* = 1.4; *green*: *τ* = 13.4 ns, *λ* = 0.7, *ν* = 1.0, *δ* = 1.0) as well as their corresponding long-time asymptotic decay (*dashed lines*). Notice that the case *τ* = 13.4 ns, *λ* = 0.7, *ν* = 1.0, *δ* = 1.0 reduces to the one-parameter Mittag-Leffler function, whose short-time behavior corresponds to the stretched exponential exp(−*t*/*τ*)^*λ*^ (86) (*black dashed line*). To see this figure in color, go online.

**Table 3:** The number of lipids for each species composing the membrane

Lipid type	Upper leaflet number	Lower leaflet number
Cholesterol	300	300
DOPC	12	12
DPPC	30	30
POPC	54	54
DGPE	12	12
POPS	18	18
PAPI	18	18
DPSM	54	54
PNSM	18	18
POSM	6	6

**Table 4:** The number of each constituent of the system

Number of water molecules	14,077
Number of Na^+^ ions	825
Number of Cl^−^ ions	161
Number of lipids	1044
Number of proteins	1

**Table 5:** Geometrical parameters of the simulated system

	Upper leaflet	Lower leaflet
Protein area	1152.12249 Å^2^	1126.52528 Å^2^
Lipid area	26,331.6 Å^2^	26,331.6 Å^2^
Total area	27,483.72249 Å^2^	27,458.12528 Å^2^
Protein X extent	26.06 Å	–
Protein Y extent	24.69 Å	–
Box Y extent	165.74 Å	–
Box X extent	165.74 Å	–
Box Z extent	109.31 Å	–

**Table 6:** Sampling time step and total length of the numerical simulations

Regimes	Sampling time step	Time length
Ballistic	20 fs	1000 ps
Subdiffusive	20 ps	3 *μ*s
Diffusive	5000 ps	100 *μ*s

**Figure 9:**
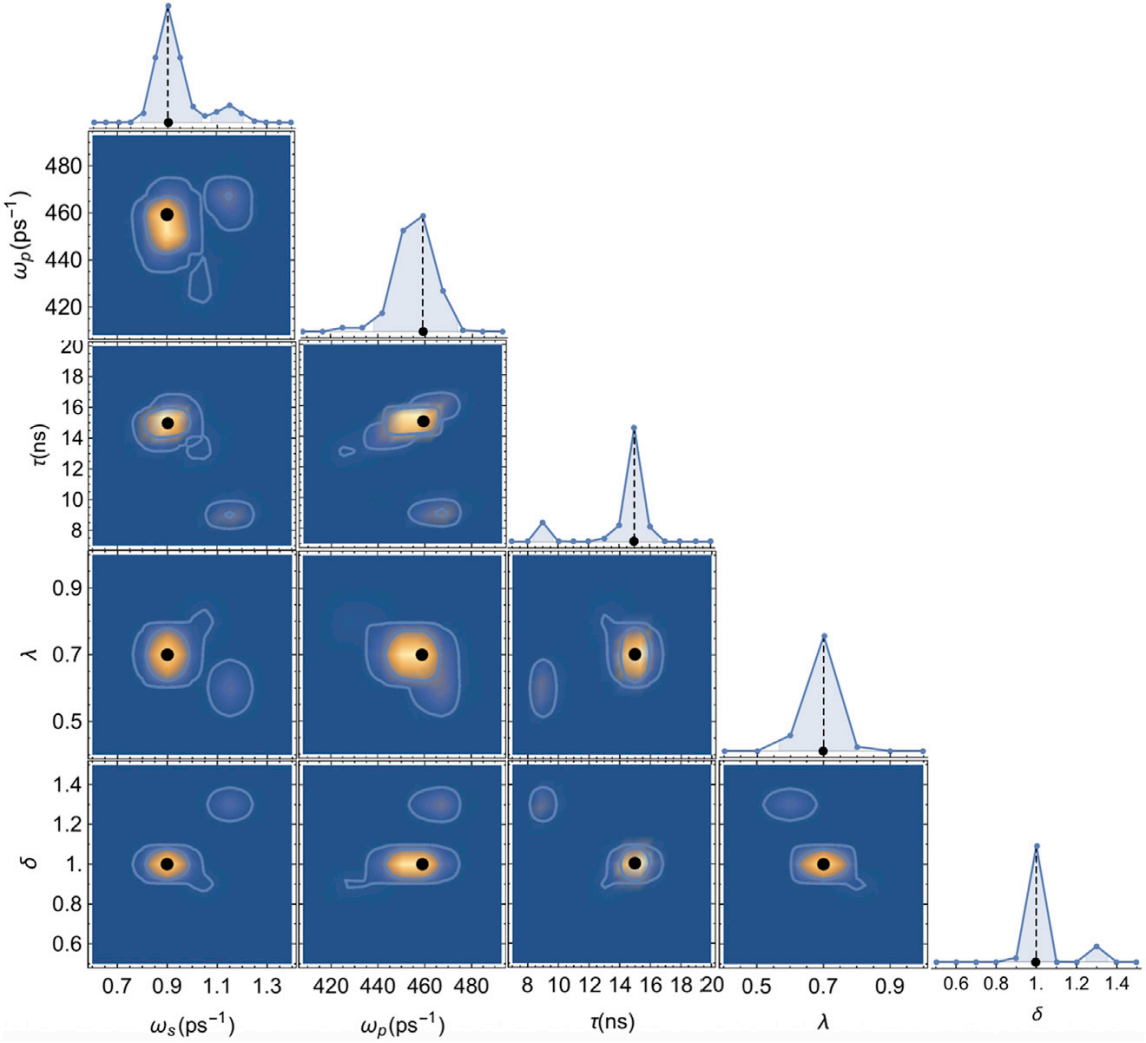
One-dimensional and two-dimensional marginal distributions for model M1. The highlighted area below the one-dimensional marginal distributions corresponds to 95%. The ellipses in the two-dimensional marginal distributions correspond to 68 and 95% confidence regions. The reduced ${\tilde{\chi }}^{2}$ is 1.02. To see this figure in color, go online.

**Table 7:** Best-fit parameters of model M1

*δ*^a^	*λ*^a^ = *δν*	*τ*^a^ (ns)	*ω*_*s*_^a^ (ps^−1^)	*ω*_*p*_^a^ (ps^−1^)
$.1.0_{0.9}^{1.3}$	$0.7_{0.55}^{0.8}$	$15.0_{8.5}^{16.5}$	$0.9_{0.8}^{1.2}$	$459_{440}^{475}$

The sub- and superscripts represent the 95% confidence intervals.

a Best-fit parameters.

## Appendix a: Asymptotic behavior of the three-parameter mittag-leffler function

The three-parameter Mittag-Leffler function is defined as (48,49)

(35) $$E_{\lambda ,\nu }^{\delta }\left(z\right)=\overset{\infty }{\underset{k=0}{\sum }}\frac{{\left(\delta \right)}_{k}}{\text{Γ}\left(\lambda k+\nu \right)}\frac{z^{k}}{k!},$$

where (*δ*)_*k*_ is the Pochhammer symbol; Γ is the Euler-Γ function; and *λ*, *ν*, and *δ* could be, in general, complex numbers but with *Re*[*λ*] > 0. The long-time expression of the three-parameter Mittag-Leffler function follows a power-law behavior given by (62)

(36) $$E_{\lambda ,\nu }^{\delta }\left(-z\right)\approx \frac{z^{-\delta }}{\text{Γ}\left(\nu -\delta \lambda \right)}-\frac{\delta z^{-\left(\delta +1\right)}}{\text{Γ}\left(\nu -\left(\delta +1\right)\lambda \right)},\;\left(\left|z\right|\gg 1\right).$$

A few different examples of Mittag-Leffler functions are illustrated in Fig. 8 together with their asymptotic behavior. The plots are given for a set of parameters satisfying the complete monotonicity condition, 0 < *λ* ≤ 1, 0 < *λδ* ≤ *ν* ≤ 1. Note that the leading term in Eq. 36 is the first one for *λδ* < *ν*, whereas for *λδ* = *ν*, it is the second one.

The plot of the three-parameter Mittag-Leffler function shown in Fig. 8 is done with the Mathematica software by implementing the integral representation of the generalized three-parameter Mittag-Leffler function as explained in the Supporting materials and methods, Section I.

## Appendix b: Numerical details

In this appendix, we report further details of the MD simulation setup. The details about the constituents of the system, the membrane composition, the geometrical parameters of the system, and the trajectory length are reported in Tables 3, 4, 5, and 6, respectively.

The whole system was prepared by using CHARMM-GUI membrane builder web server (87). The simulations were performed through the GROMACS program suite (88, 89, 90, 91), version 2016.3, in double precision, using a Martini force field (65, 66, 67) with the leap-frog integration algorithm (92) and an integration time step of 20 fs. Electrostatic interactions were calculated using the reaction-field scheme, and a cutoff of 1.1 nm for both the electrostatic and van der Waals interactions was used.

We followed the minimization and equilibration protocol of CHARMM-GUI previously used in our lab on analogous systems (93):

1 Minimization:
a 2.5 × 10^5^ steps of steepest descent minimization with a soft-core potential on Lennard-Jones and Coulomb interactions
b 2.5 × 10^4^ steps of steepest descent minimization.
2 The equilibration is carried out in NPT ensemble using the Berendsen barostat (93) (reference pressure 1 bar, coupling constant 5 ps, and compressibility 3 × 10^−4^ bar^−1^) and the velocity rescale thermostat (94) (reference temperature 310 K, coupling constant 1 ps). The procedure is split into five simulation steps:
a 5 × 10^5^ steps (time step 0.002 ps, trajectory length 1000 ps) with position restraint on the headgroups (200 kJ/mol/nm^2^) and the protein (1000 kJ/mol/nm^2^);
b 2 × 10^5^ steps (time step 0.005 ps, trajectory length 1000 ps) with position restraint on the headgroups (100 kJ/mol/nm^2^) and the protein (500 kJ/mol/nm^2^);
c 1 × 10^6^ steps (time step 0.010 ps, trajectory length 10,000 ps) with position restraint on the headgroups (50 kJ/mol/nm^2^) and the protein (250 kJ/mol/nm^2^);
d 5 × 10^5^ steps (time step 0.015 ps, trajectory length 7500 ps) with position restraint on the headgroups (20 kJ/mol/nm^2^) and the protein (100 kJ/mol/nm^2^); and
e 5 × 10^5^ steps (time step 0.020 ps, trajectory length 7500 ps) with position restraint on the headgroups (10 kJ/mol/nm^2^) and the protein (50 kJ/mol/nm^2^).

Three production runs were carried out in the isothermal–isobaric ensemble (NPT) using the Parrinello-Rahman barostat (96) (reference pressure 1 bar, coupling constant 15 ps, compressibility 3 × 10^−4^ bar^−1^) and the velocity rescale thermostat (95) (reference temperature 310 K, coupling constant 1 ps). Different sampling times were used to fully resolve the different dynamical regimes (see Table 6). From these simulations, we extracted the protein’s center-of-mass trajectories. The lateral MSD of the center of mass was calculated through the GROMACS tool analyze with the flag -msd. The maximal time lag considered corresponds to 10% of the trajectory length.

To give an idea of the computational effort, a 10-*μ*s-long trajectory carried out on a 48-core machine (Intel Xeon(R) CPU E5-2687W v4) requires 4 days (i.e., 2.5 *μ*s/day).

## Appendix c: Data analysis

To evaluate best-fit values of the model parameters and their uncertainties, we used a Bayesian approach with a flat (uninformative) prior distribution for the parameters (72), i.e., all the parameter values are assumed to be equally probable before analyzing the data.

In accordance with Bayes’s theorem, the posterior distribution of a model described by the parameters ***θ***, given the measured data **d**, reads

(37) $$P\left(\theta |d\right)=\frac{L\left(d|\theta \right)\text{Π}\left(\theta \right)}{E\left(d\right)},$$

where

(38) $$L\left(d|\theta \right)=\frac{1}{Z}{\text{e}}^{-\chi^{2}/2}=\frac{1}{Z}\text{exp}\left[-\frac{1}{2}\overset{N}{\underset{i=1}{\sum }}{\left(\frac{m\left(\theta ,i\right)-d_{i}}{\sigma_{i}}\right)}^{2}\right],$$

is the likelihood of the MSD data **d** = {*d*_*i*_}, given the MSD model *m*(***θ***, *i*) calculated for the parameters ***θ***, with *i* being the index running over the points of the data set of size *N* and *σ*_*i*_ the error of the data. *Z* is a normalization constant that guarantees that $\int L\left(d|\theta \right)d^{5}\theta$ = 1. The evidence of the data, *E*(**d**), amounts to a normalization constant whose value is fixed, and Π(***θ***) is the prior distribution of parameters, which is assumed to be uniform (72).

To obtain an accurate estimation of the subdiffusive-to-Brownian dynamics crossover, many microscopic fluctuations must be observed. Thus, the relative error on the observed MSD has been estimated as the reciprocal of the square root of the number of observed fluctuations and so as (*τ*^∗^/*T_o_*)^1/2^, where *τ*^∗^ is the characteristic time of the fully Brownian dynamics recovering, ∼400 ns, and *T*_*o*_ is the observation time (i.e., the largest trajectory length).

The posterior distribution of the model is sampled over a regular grid of the parameter space:

M1: *ω*_*s*_ ∈ [0.6, 1.4] ps^−1^, *ω*_*p*_ ∈ [408, 500] ps^−1^, *τ* ∈ [7000, 20,000] ps, *λ* ∈ [0.4, 1.0], and *δ* ∈ [0.51, 1.5]. Number (#) of grid points: ${\#}_{\omega_{s}}$ = 17, ${\#}_{\omega_{p}}$ = 11, #_*τ*_ = 14, #_*λ*_ = 7, #_*δ*_ = 11; and
M2: *ω*_*s*_ ∈ [0.7, 1.3] ps^−1^, *ω*_*p*_ ∈ [409.5, 491.4] ps^−1^, *τ* ∈ [10,000, 17,000] ps, and *λ* (= *ν*) ∈ [0.68, 0.76]. Number (#) of grid points: ${\#}_{\omega_{s}}$ = 25, ${\#}_{\omega_{p}}$ = 25, #_*τ*_ = 36, #_*λ*_ = 17.

The expectation value of each parameter is estimated by the maximum of the one-dimensional marginalized posterior distribution, i.e., after marginalizing P over all but one parameter in turn (Fig. 3 for model M2 and Fig. 9 for model M1). The parameters confidence intervals correspond to the 95% of the area below the one-dimensional marginalized posterior distribution starting from the maximum. The best-fit parameters are summarized in Table 2 for model M2 and Table 7 for model M1. To estimate the correlations between the parameters of the model, the two-dimensional marginal distribution for each pair of parameters (obtained by marginalizing over *n* − 2 parameters, where *n* is the number of free parameters) is calculated as well (Fig. 3 for model M2 and Fig. 9 for model M1).

## Supporting material

Document S1. Supporting materials and methods, Figs. S1–S13, and Tables S1 and S2

Document S2. Article plus supporting material
