# Supplementary material for: Subdiffusive-Brownian crossover in membrane proteins: a generalized Langevin equation-based approach
Source: Biophys J. 2021 Sep 28;120(21):4722–37. doi: 10.1016/j.bpj.2021.09.033 (PMC8595736; doi:10.1016/j.bpj.2021.09.033)
Supplement: Document S1. Supporting materials and methods, Figs. S1–S13, and Tables S1 and S2 [file mmc1.pdf]

Biophysical Journal, Volume *120*

Supplemental information

Subdiffusive-Brownian crossover in membrane proteins: a generalized Langevin equation-based approach

Loris Di Cairano, Benjamin Stamm, and Vania Calandrini

Supplementary Information:
Subdiffusive-Brownian crossover in membrane proteins: a Generalized Langevin Equation-based approach

Loris Di Cairano*

Department of Physics, Faculty of Mathematics, Computer Science and Natural Sciences, Aachen University, 52062 Aachen, Germany and Computational Biomedicine, Institute of Neuroscience and Medicine INM-9 and Institute for Advanced Simulations IAS-5, Forschungszentrum Jülich, 52428 Jülich, Germany

Benjamin Stamm†

Applied and Computational Mathematics, Department of Mathematics, RWTH Aachen University, Aachen, Germany

Vania Calandrini‡

Computational Biomedicine, Institute of Neuroscience and Medicine INM-9 and Institute for Advanced Simulations IAS-5, Forschungszentrum Jülich, 52428 Jülich, Germany

I. NUMERICAL METHODS TO CALCULATE THE THREE-PARAMETER MITTAG-LEFFLER FUNCTIONS

In this section, we sketch the two numerical methods we have implemented in Mathematica to evaluate and visualize the three-parameter Mittag-Leffler function. Mathematica indeed provides by default only the one and the two-parameter Mittag-Leffler functions. Alternatively, one could adopt the Matlab routine provided by Garrappa, which implements the three-parameter Mittag-Leffler function [1, 2].
The first method is based on the integral representation of the generalized 3-parameter Mittag-Leffler, whereas the second one is based on the numerical computation of the inverse Laplace transform of the generalized three-parameter Mittag-Leffler expression in the Laplace space. Note that we do not intend to give an exhaustive explanation of the three-parameter Mittag-Leffler function and related topics; in contrast, we will give a brief overview and refer to the relevant literature [2–10] for details. Let us start with recalling the generalized three-parameter Mittag-Leffler function:

$$e^{\delta}_{\lambda,\nu}\left(\frac{t}{\tau}\right) = \left(\frac{t}{\tau}\right)^{\nu-1} E^{\delta}_{\lambda,\nu}\left[-\left(\frac{t}{\tau}\right)^{\lambda}\right]. \tag{1}$$

In [3, 9] it has been shown that the function defined in (1) can be rewritten in form of an integral as

$$e^{\delta}_{\lambda,\nu}\left(\frac{t}{\tau}\right) := \int_0^\infty p^{\delta,\tau}_{\lambda\nu}(f) e^{-ft} df, \tag{2}$$

where the distribution function $p^{\delta,\tau}_{\lambda,\nu}$ is given by

$$\begin{aligned} p^{\delta,\tau}_{\lambda\nu}(f) &= \frac{\tau(f\tau)^{\lambda\delta-\nu}\sin\left[\pi(\nu-\lambda\delta)+\delta\theta^{\tau}_{\lambda}(f)\right]}{\pi\left[(f\tau)^{2\lambda}+2(f\tau)^{\lambda}\cos(\pi\lambda)+1\right]^{\delta/2}}, \\ \theta^{\tau}_{\lambda}(f) &= \arg\left[(-f\tau)^{\lambda}+1\right], \end{aligned} \tag{3}$$

for $0<\lambda\leq 1$ and $0<\lambda\delta\leq\nu\leq 1$.

In the first approach one numerically evaluates the integral in Eq. (2) by using Eq. (3) for a given set of parameters. This provides the generalized three-parameters Mittag-Leffler function $e^{\delta}_{\lambda,\nu}(t/\tau)$. Then, one can multiply the result by $(t/\tau)^{1-\nu}$ to obtain the 3-parameter Mittag-Leffler function $E^{\delta}_{\lambda,\nu}\left[-(t/\tau)^{\lambda}\right]$.

*Electronic address: l.di.cairano@fz-juelich.de
†Electronic address: best@acom.rwth-aachen.de
‡Electronic address: v.calandrini@fz-juelich.de

An alternative approach is based on the use of the inverse Laplace transform (ILT). It consists in employing a numerical method for inverting the Laplace transform of the function (1), which is given by (see [11]):

$$\mathscr{L}[e^{\delta}_{\lambda,\nu}](s) := \frac{1}{\tau^{\nu-1}} \frac{s^{\delta\lambda-\nu}}{(s^{\lambda}+\tau^{-\lambda})^{\delta}}. \tag{4}$$

For this method, one can use the Mathematica ILT function developed by Horváth I. et al. in Ref. [12, 13].
The numerical inversion of Eq.(4) provides the generalized three-parameter Mittag-Leffler function. Again, the 3-parameter Mittag-Leffler function can be obtained by multiplying the result by $(t/\tau)^{1-\nu}$.

Figure 1 visualizes the generalized three-parameter Mittag-Leffler function for $\lambda = 0.4$ and different values of ν with $\delta = \nu/\lambda$ and one can observe that the two methods produce values that are not distinguishable by eye.

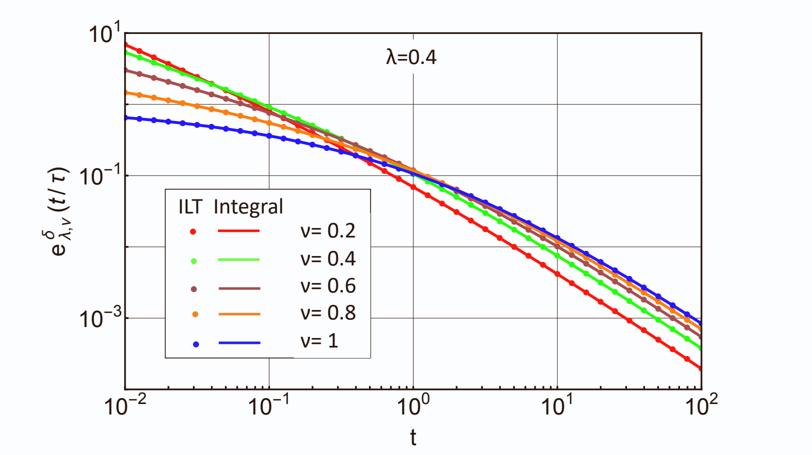

FIG. 1: A variety of generalized three-parameter Mittag-Leffler functions calculated through the discrete ILT algorithm (dots) and the integral representation (lines) for different parameter values ($\lambda = 0.4$, $\nu = \{0.2, 0.4, 0.6, 0.8, 1\}$, and $\delta = \nu/\lambda$).

II. SURVEY OF THE THREE PARAMETERS MITTAG-LEFFLER FUNCTIONS WITH THEIR SPECTRAL DISTRIBUTIONS

In this section, we present an investigation on the qualitative behavior of the three-parameter Mittag-Leffler function $E^{\delta}_{\lambda,\nu}[-(t/\tau)^{\alpha}]$, the generalized three-parameter Mittag-Leffler function $e^{\delta}_{\lambda,\nu}(t/\tau)$ as well as the spectrum $p^{\delta,\tau}_{\lambda,\nu}(f)$. The plots are drawn using the integral representation method briefly explained in the previous section. The parameter τ is fixed to 1, whilst λ, ν and δ are varied in the intervals of interest for this work, i.e. $0 < \lambda \leq 1$, $0 < \lambda\delta = \nu \leq 1$. Specifically, in Figs. 2 and 3 we varied ν fixing λ, in Figs. 4 and 5 we varied λ fixing ν and in Figs. 6 and 7 we varied $\lambda(=\nu)$ so that δ equals 1.

With the constraints imposed in our parameters, the analysis reported in this section indicates that ν fixes the short time behavior of $e^{\delta}_{\lambda,\nu}$ (Figure 5,a,d); when ν is fixed, the high frequency tails of $p^{\delta}_{\lambda\nu}$ collapse on the same curve independently from the values of $\lambda(\delta)$ (Figure 5,c,f). For $\nu \to 1$ the small relaxation times (high frequencies f) contribution decreases and vice versa for $\nu \to 0$ (Figure 3,a,d,c,f). The parameter λ fixes the form of the low frequencies tail of $p^{\delta}_{\lambda\nu}$ (long time behavior of $e^{\delta}_{\lambda,\nu}$), but the curves are shifted by a constant depending on the value of δ (notice the log-log scale) (Figure 3,a,d,c,f). For $\lambda \to 1$ the large relaxation times contribution decreases and vice versa for $\lambda \to 0$ (Figure 5,a,d,c,f). This is in agreement with the asymptotic behavior of $e^{\delta}_{\lambda,\nu}$ for $t \to \infty$ reported in the literature that, for $0 < \lambda\delta = \nu \leq 1$, is $\sim t^{-1-\lambda}$. Finally, for $\delta = 1$ the gradient of the distribution is zero at $1/\tau$ (Figure 7,c)), whilst for $\delta < 1$ the stationary point moves to higher frequencies and vice versa for $\delta > 1$ (Figures 3,c,f and 5,c,f).

III. USING MITTAG-LEFFLER FUNCTIONS TO CAPTURE THE MEMORY KERNEL OF LIQUID ARGON

In order to check the reliability of the Mittag-Leffler functions in capturing small deviations from the ideal Dirac $\delta-$function kernel predicted for a truly Brownian system, we analyzed in silico MSD data of pure Argon and we compared the VACF predicted by the model with the numerical counterpart.
MD simulations have been run using Rahman's setup [14], consisting of a system of 864 particles interacting through a Lennard-Jones potential $V(r) = 4\varepsilon[(\sigma/r)^{12} - (\sigma/r)^{6}]$, $\varepsilon/k_B = 120$K and $\sigma = 0.34$ nm, in a cubic box of side $L = 10.229\sigma$ (density=1.374

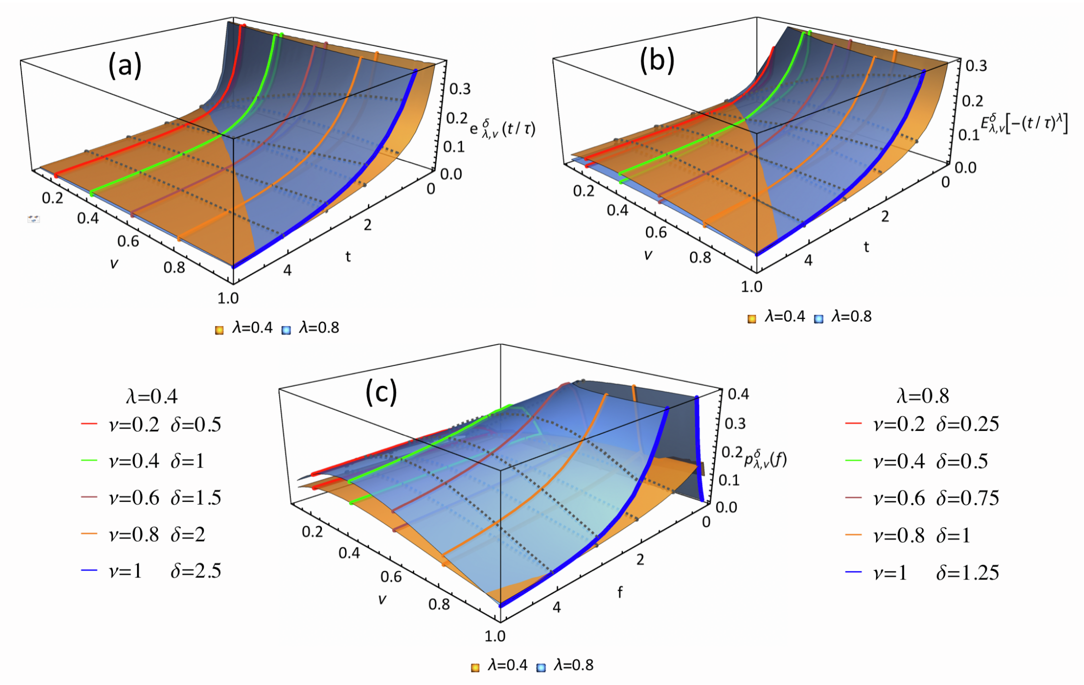

FIG. 2: 3D illustration of the functions $e^{\delta}_{\lambda,\nu}$ (a), $E^{\delta}_{\lambda,\nu}$ (b) and $p^{\delta}_{\lambda\nu}$ (c) for $\lambda = 0.4$ and $\lambda = 0.8$ as a function of ν and, respectively, t for (a) and (b), f for (c). δ is fixed to the value ν/λ. The curves corresponding to some specific ν values are highlighted according with the color code in the label.

$^{*}\omega_s$ (ps^{-1})	ω_p (ps^{-1})	τ (ps)	$\lambda(=\nu)$	$^{*}\delta$	$^{**}\omega_0$	$^{**}D_\infty$ (cm^2 s^{-1})
0	8.2	0.12	0.84	1	8.27	$2.40 \cdot 10^{-5}$

TABLE I: Best fit parameters for MSD data of Ar. The superscript * indicates the parameters kept fixed during the fitting. The superscript ** indicates the parameters derived from the fitted ones.

gcm^{-3}) at temperature $T = 94.4$ K, with periodic boundary conditions. Lennard-Jones interactions cutoff was set to 1.2 nm. The integration time step was 1 fs and the trajectory length 1 ns. The minimum time lag considered for MSD and VACF calculations was 10 fs, and the maximum 100 ps.
MSD data have been analyzed according to Eq.(35) in the main text by setting $\omega_s = 0$, so that the overall Ar friction is described by the generalized 3-parameter Mittag-Leffler function alone

$$\zeta_{Ar}(t) := M\omega_0^2 \left(\frac{t}{\tau}\right)^{\nu-1} E^{\delta}_{\lambda,\nu}\left[-\left(\frac{t}{\tau}\right)^{\lambda}\right]. \tag{5}$$

With this setting, $1/\omega_0$ corresponds to the end of the ballistic regime and the generalized 3-parameter Mittag-Leffler function accounts for the deviation from the Dirac-$\delta-$function. The fit to MSD data is shown in the top left panel of Figure 8 and the resulting parameters are summarized in Table I. Notice that $1/\omega_0 \approx \tau(= 0.12$ ps), which is the observation time scale of the distribution of relaxation times leading to diffusion. The integral of the memory kernel ζ_{Ar}/M is given by $\omega_p = 8.2$ ps^{-1} (which corresponds to $\omega_0^2\tau$). This value is in fair agreement with the estimation of 7.33 ps^{-1} reported by Kneller et al. in [15]. The overall shape of the memory function as well compares quite well with the one reported by Kneller et al. (see bottom panel of Fig. 8 and Fig.1 in [15]).
The comparison between the VACF predicted by the model using the MSD best fit parameters and the one from MD simulation is shown in the top right panel of Fig. 8. A persistent asymptotic negative correlation $\sim -t^{-1.84}$ is predicted. Finally, we note that the Brownian diffusion coefficient given by $k_BT/(M\omega_p)$ is in good agreement with the value reported by Rahman [14].

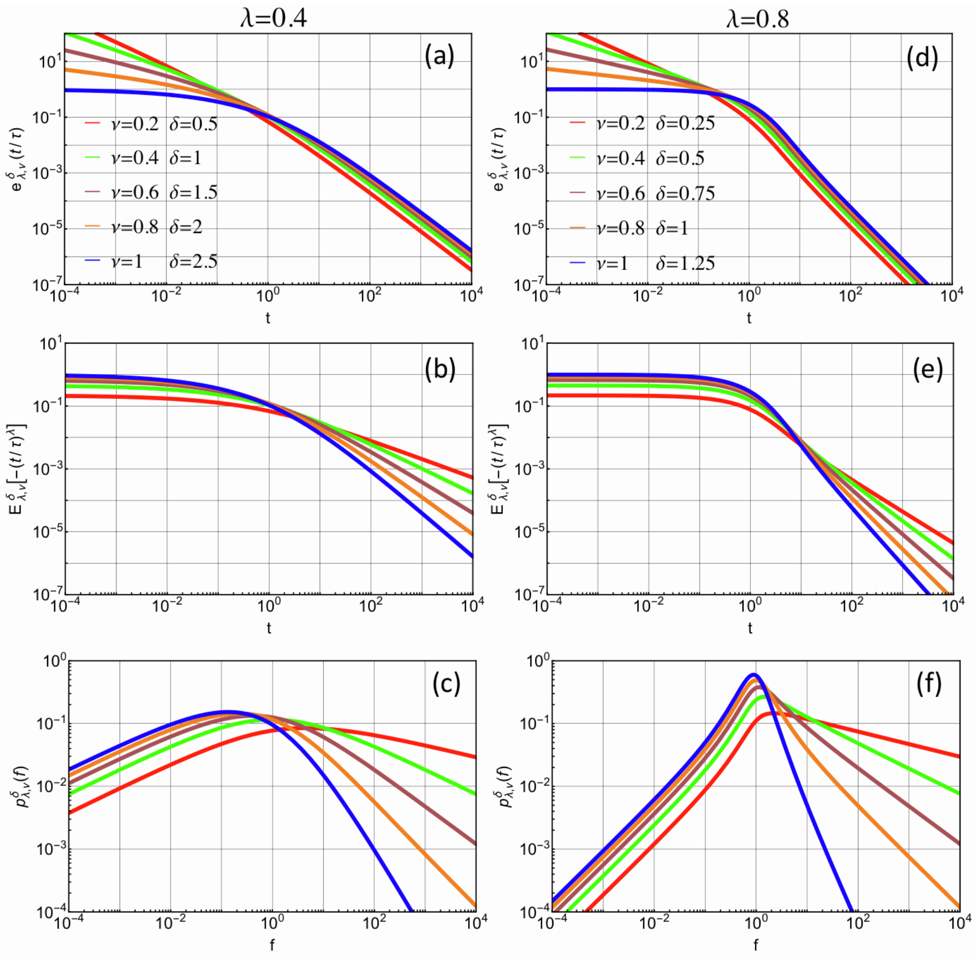

FIG. 3: 2D illustration in log-log scale of the functions $e^{\delta}_{\lambda,\nu}$ (a,d), $E^{\delta}_{\lambda,\nu}$ (b,e) and $p^{\delta}_{\lambda\nu}$ (c,f) for $\lambda = 0.4$ (a,b,c) and $\lambda = 0.8$ (d,e,f) for the ν values highlighted in Fig. 2.

IV. APPLICATION OF THE MODEL TO SIMPLE BILAYERS

In this section, we present the results of the model proposed in the main text when applied to intermediary control systems. As a control, we used in-silico bilayers composed of

(1) 100% POPC (POPC100).

(2) M2 receptor (3uon) in 100% POPC (M2-POPC100)

(3) M2 receptor (3uon) in 50% POPC/50% Cholesterol (M2-POPC50/CHOL50)

The simulation setups are as follow:

species	POPC100	M2-POPC100	M2-POPC50/CHOL50
3uon	0	1	1
POPC	660	1046	576
CHOL	0	0	576
water	5675	15355	13762
ions	135	369	325

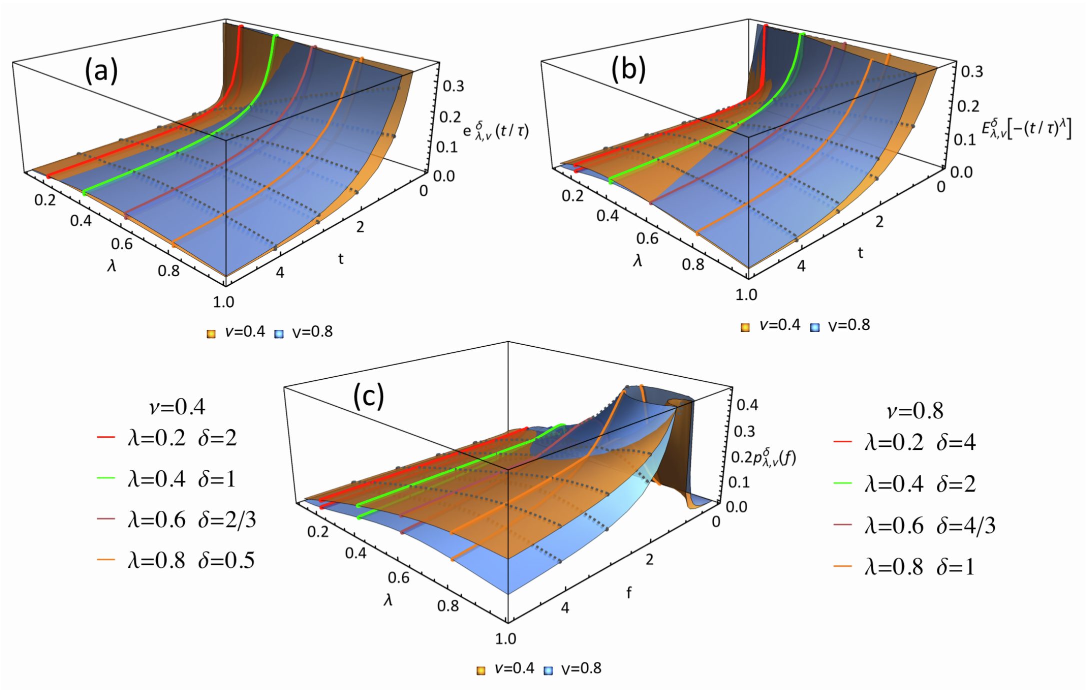

FIG. 4: 3D illustration of the functions $e^{\delta}_{\lambda,\nu}$ (a), $E^{\delta}_{\lambda,\nu}$ (b) and $p^{\delta}_{\lambda\nu}$ (c) for $\nu = 0.4$ and $\nu = 0.8$ as a function of λ and, respectively, t for (a) and (b), f for (c). δ is fixed to the value ν/λ. The curves corresponding to some specific λ values are highlighted according with the color code in the label.

The simulation protocol is the same used for the mixed membrane reported in the main text.
Gaussianity of lipids in protein poor conditions and up to $\sim 20\%$ of cholesterol concentration has been proved in [16–18]. Here we report the Gaussianity test for the protein diffusing in pure POPC and in the two component bilayer POPC/Cholesterol 50:50. The test, which supports the Gaussianity hypothesis (see Fig. 9), is based on the calculation of the cumulative distribution function for r^2 displacements as explained in the main text.
The model has then been fitted to the MSD of an average representative lipid for systems (1) and to the MSD of the receptor for systems (2) and (3) (see Fig.10). The best fit parameters and the parameters derived from them are reported in Table II. Although not exhaustive, the analysis of these simple systems proves the capability of the model to describe different membrane systems and allows rationalizing some of their general features. The model suggests that the end of the ballistic regime (ω_s) occurs at shorter times for the lipids than for protein. In the presence of cholesterol, protein's transition from ballistic to (sub)diffusion is faster. The same trend is observed for the characteristic frequency of the transient harmonic potential, ω_0. ω_0 is larger for the lipids than for protein. Cholesterol increases the frequency of the confining potential acting on the protein. The maximum of the relaxation rates spectrum, $1/\tau$, occurs at longer times for the protein than for lipids. Cholesterol shifts the maximum of the protein relaxation spectrum to higher frequencies and reduces its width; λ actually increases when the protein diffuses in the presence of cholesterol. It is interesting to note that simultaneously, the transient confinement of the protein increases (because of the ω_0 increase).

V. MSD AND VACF OF THE MIXED MEMBRANE

In Fig. 12 we show the MSD data of an average representative lipid in the NEAR and FAR shell as defined in the main text. For comparison, protein's MSD is shown as well. Notice that the model cannot be applied to the lipids of the mixed membrane since sizable deviations are observed from a truly Gaussian process (see Fig. 11).
For time lags larger than $\sim$ 400 ns NEAR and FAR lipids essentially recover the Brownian regime with the same diffusion coefficient. The black line in the plot is calculated using the diffusion coefficient as obtained from the numerical derivative of the MSD for time lags larger than 400 ns ($\sim 4.08\ \mu\text{m}^2\text{s}^{-1}$). See Fig.5 in the main text for the evolution over time of the diffusion

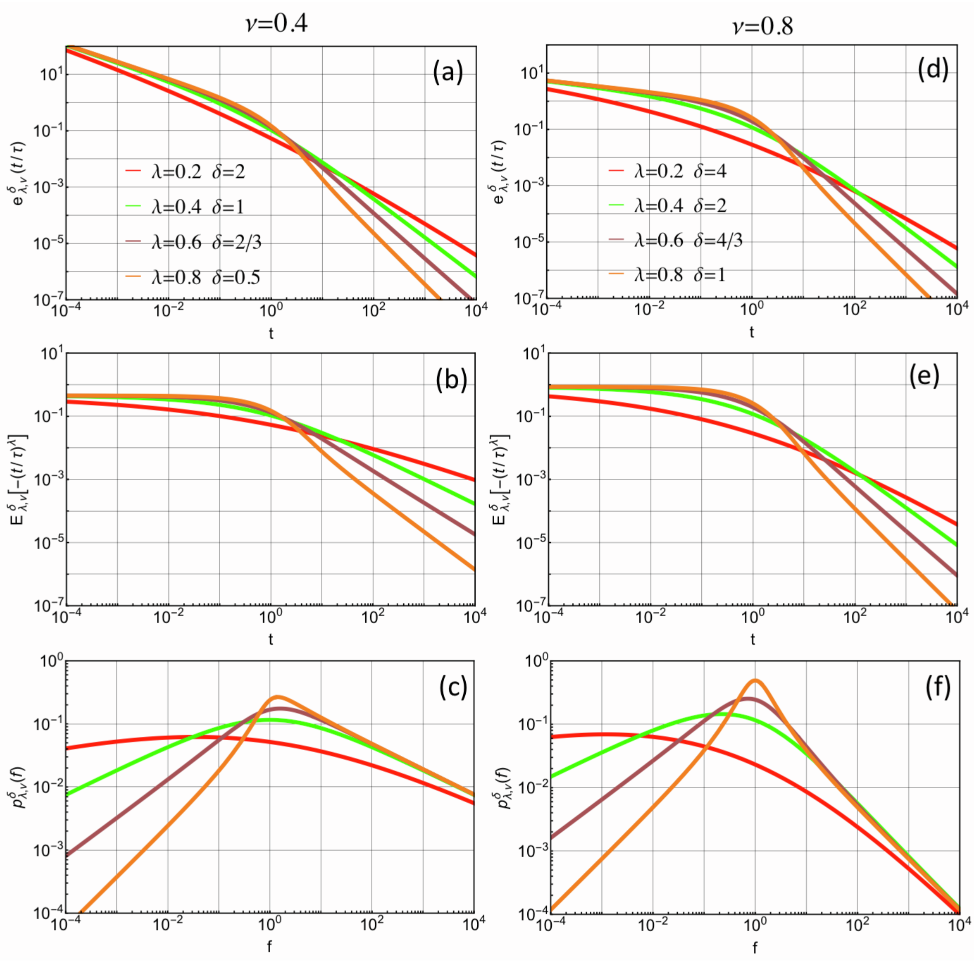

FIG. 5: 2D illustration in log-log scale of the functions $e^{\delta}_{\lambda,\nu}$ (a,d), $E^{\delta}_{\lambda,\nu}$ (b,e) and $p^{\delta}_{\lambda\nu}$ (c,f) for $\nu = 0.4$ (a,b,c) and $\nu = 0.8$ (d,e,f) for the λ values highlighted in Fig. 4.

Parameters	POPC100	M2-POPC100	M2-POPC50/CHOL50
ω_s (ps^{-1})	2.5	0.6	1
ω_p (ps^{-1})	46	8.5	70
τ (ps)	250	3900	1800
$\lambda (= \nu)$	0.5	0.3	0.7
$^{**}\omega_0$ (ps^{-1})	0.43	0.04	0.2
$^{**}D_\infty$ ($\mu m^2 s^{-1}$)	59.8	7.2	0.9

TABLE II: Best fit parameters of the model fitted to the MSD data from MD simulations of pure POPC (POPC100), POPC:Cholesterol 50:50 (POPC50/Chol50), M2 in pure POPC (M2-POPC100), M2 in POPC:Cholesterol 50:50 (M2-POPC50/Chol50). Superscript ** indicates the parameters derived from the fitted ones. δ is fixed to 1.

coefficient calculated through the numerical derivative of the MSD. The absolute VACFs are reported as well, together with the one of the protein (Figure 13). The asymptotic behavior of the protein VACF predicted by the model using the MSD best fit parameters is shown in the same plot for comparison. The power law decay of the lipids VACF is quite similar to the one of the protein. Notice that, the negative correlations of the VACFs seem to persist for shorter time lags in the case of NEAR lipids ($\sim$ 5 ps) compared to FAR lipids and protein ($\sim$ 15 ps).

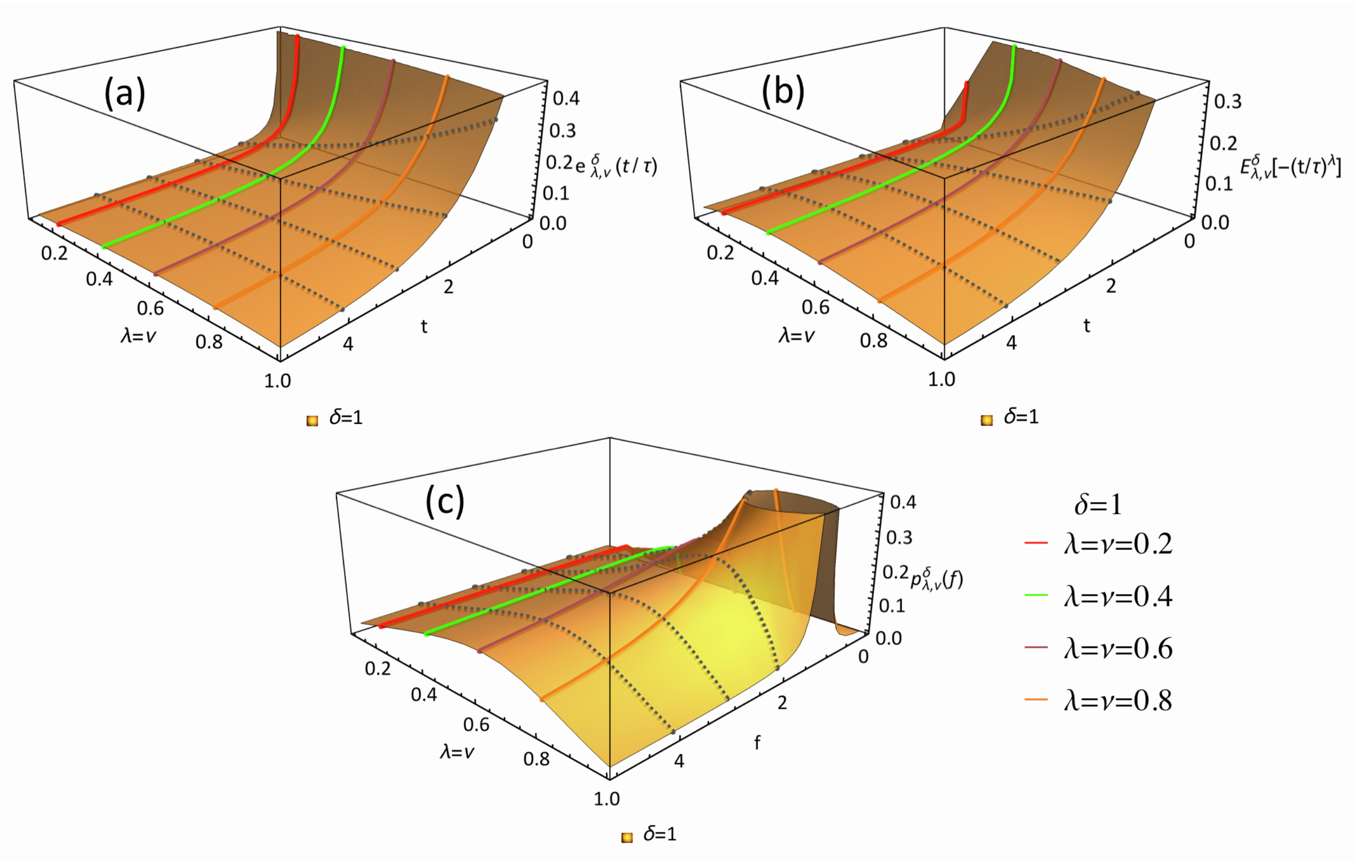

FIG. 6: 3D illustration of the functions $e^{\delta}_{\lambda,\nu}$ (a), $E^{\delta}_{\lambda,\nu}$ (b) and $p^{\delta}_{\lambda\nu}$ (c) for $\delta = 1$ as a function of $\lambda (= \nu)$ and, respectively, t for (a) and (b), f for (c). The curves corresponding to some specific $\lambda (= \nu)$ values are highlighted according with the color code in the label.

[1] *Garappa's routine for the implementation of the 3-parameter Mittag-Leffler function*, `https://www.mathworks.com/matlabcentral/fileexchange/48154-the-mittag-leffler-function`, [Online].
[2] R. Garrappa, SIAM Journal on Numerical Analysis **53**, 1350 (2015).
[3] A. Giusti, I. Colombaro, R. Garra, R. Garrappa, F. Polito, M. Popolizio, and F. Mainardi, Fractional Calculus and Applied Analysis **23**, 9 (2020).
[4] A. Giusti, Communications in Nonlinear Science and Numerical Simulation **83**, 105114 (2020).
[5] V. Kiryakova, Computers & Mathematics with Applications **59**, 1885 (2010).
[6] R. Gorenflo, A. A. Kilbas, F. Mainardi, S. V. Rogosin, et al., *Mittag-Leffler functions, related topics and applications* (Springer, 2014), 1st ed.
[7] R. Garrappa and M. Popolizio, Journal of Computational and Applied Mathematics **235**, 1085 (2011).
[8] R. Garrappa and M. Popolizio, Advances in Computational Mathematics **39**, 205 (2013).
[9] F. Mainardi and R. Garrappa, Journal of Computational Physics **293**, 70 (2015).
[10] F. Mainardi, Entropy **22**, 1359 (2020).
[11] T. Sandev, Ž. Tomovski, and J. L. Dubbeldam, Physica A: Statistical Mechanics and its Applications **390**, 3627 (2011).
[12] G. Horváth, I. Horváth, S. A.-D. Almousa, and M. Telek, Performance Evaluation **137**, 102067 (2020).
[13] *GitHub Repository with inverse Laplace transform codes*, `https://github.com/ghorvath78/iltcme/blob/master/mathematica_ilt.nb`, [Online].
[14] A. Rahman, Physical Review **136**, A405 (1964).
[15] G. R. Kneller and G. Sutmann, The Journal of Chemical Physics **120**, 1667 (2004).
[16] G. R. Kneller, K. Baczynski, and M. Pasenkiewicz-Gierula, The Journal of chemical physics **135**, 141105 (2011).

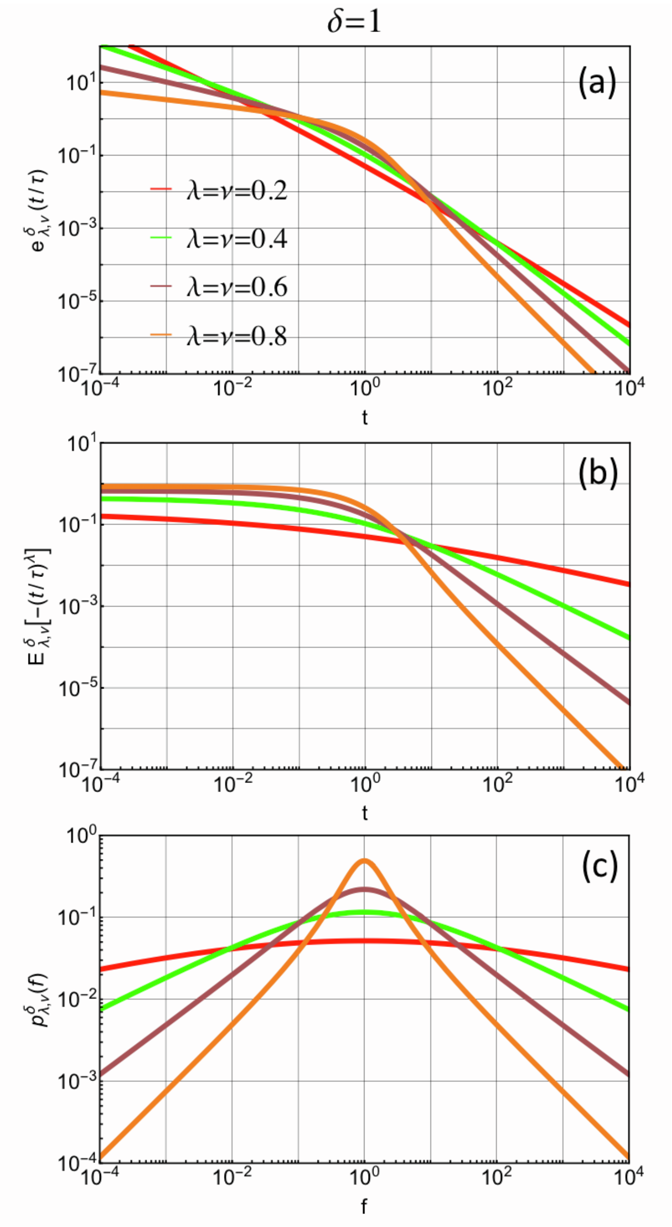

FIG. 7: 2D illustration in log-log scale of the functions $e^{\delta}_{\lambda,\nu}$ (a), $E^{\delta}_{\lambda,\nu}$ (b) and $p^{\delta}_{\lambda\nu}$ (c) for $\delta = 1$ and the $\lambda(= \nu)$ values highlighted in Fig.6.

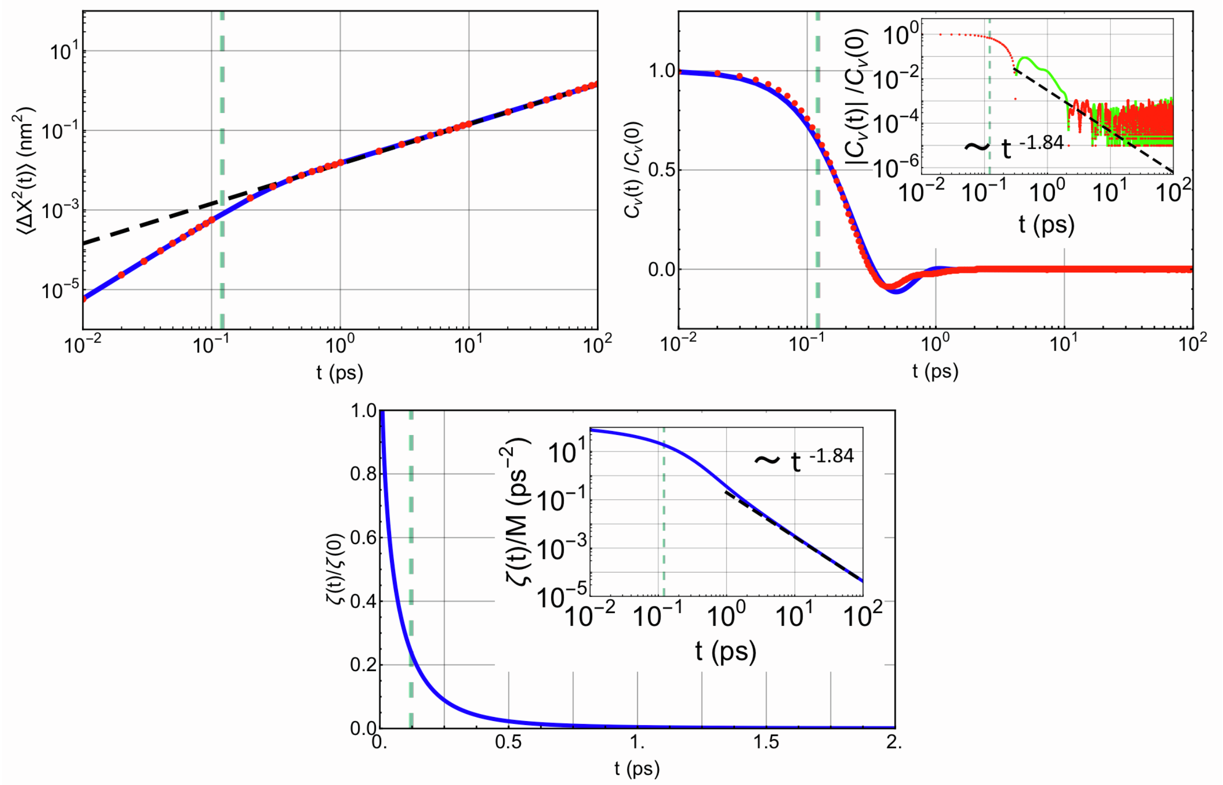

FIG. 8: Ar analysis. Top left panel: MSD from MD simulation (red points) versus fitted model (blue line). The dashed black line corresponds to the asymptotic Brownian regime. Top right panel: Comparison between VACF data from MD simulation (red points) and calculated model (blue line). Inset: Absolute VACF from MD simulation in log-log scale (red points indicates original positive values and green line negative values) together with the asymptotic behavior predicted by the model (black dashed line). Bottom panel: Normalized memory function predicted by the model. Inset: non-normalized memory function in log-log scale together with the asymptotic behavior (black dashed line). In all of the panels the dashed vertical lines in dark green indicate $1/\omega_0 \approx \tau$.

[17] J.-H. Jeon, H. M.-S. Monne, M. Javanainen, and R. Metzler, Physical Review Letters **109**, 188103 (2012).
[18] J.-H. Jeon, M. Javanainen, H. Martinez-Seara, R. Metzler, and I. Vattulainen, Physical Review X **6**, 021006 (2016).

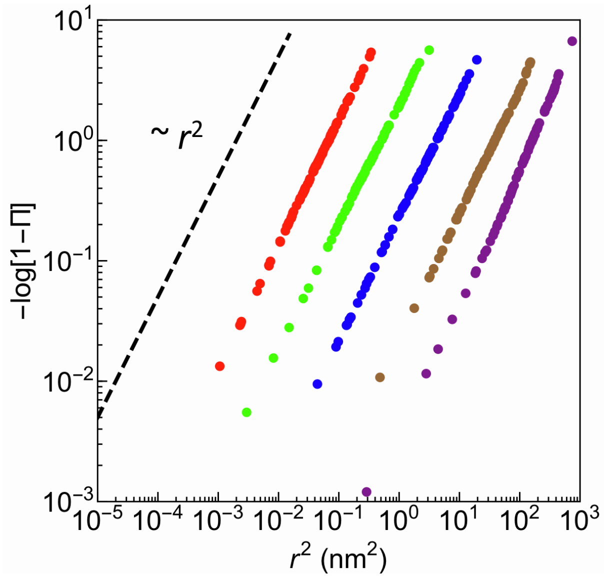

(a)

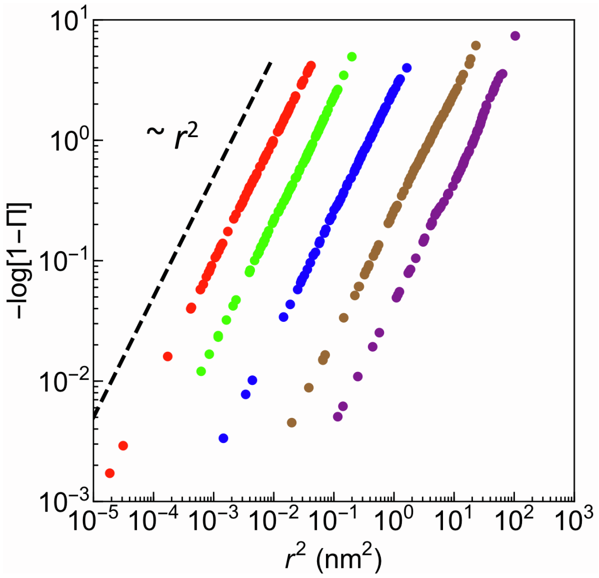

(b)

FIG. 9: Cumulative distribution $\Pi(r^2, \Delta)$ for receptor M2 diffusing in pure POPC (a) and in POPC/Chol 50:50 (b) with $\Delta = 1, 10, 100, 1000, 5000$ ns (from left to right). The dashed line corresponds to a theoretical curve proportional to r^2.

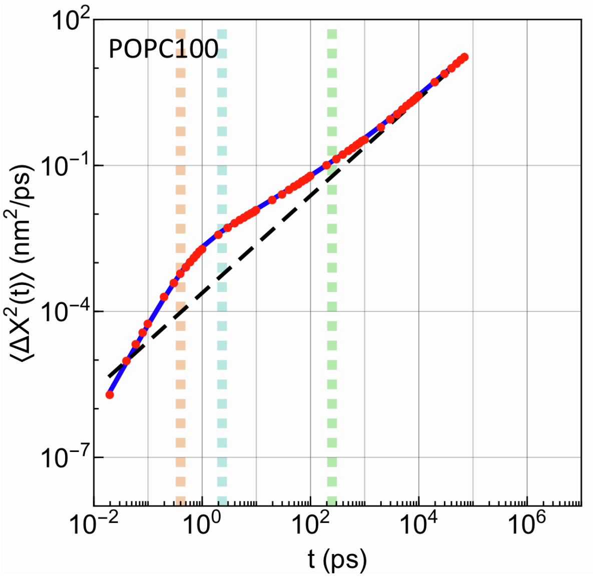

(a)

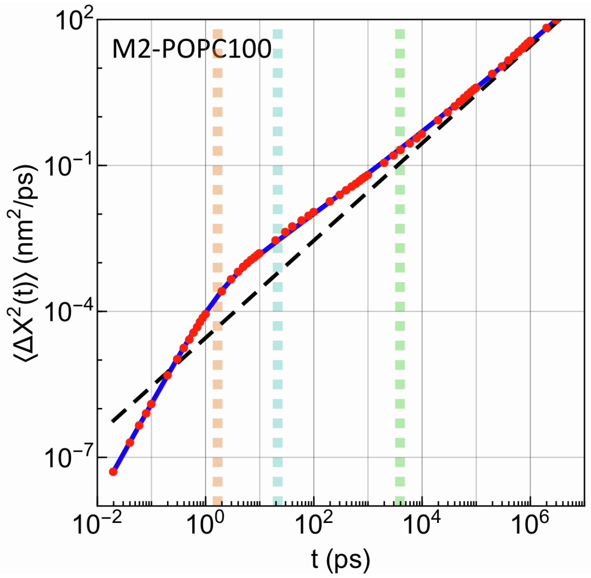

(b)

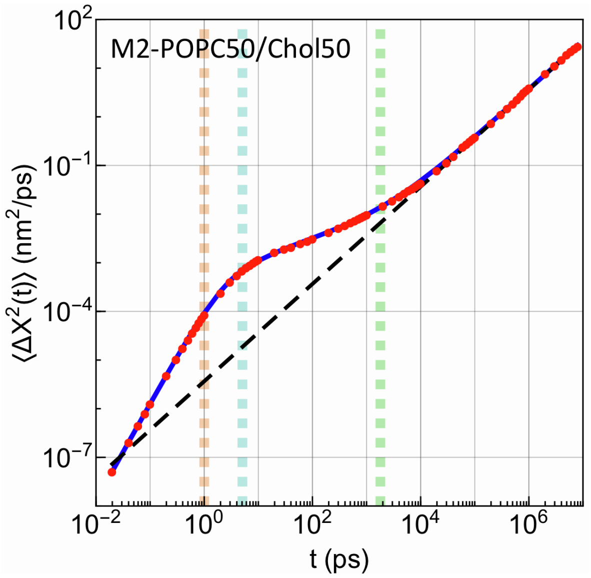

(c)

FIG. 10: MSD data from MD simulations of pure POPC (POPC100) (a), M2 in pure POPC (M2-POPC100) (b), M2 in POPC:Cholesterol 50:50 (M2-POPC50/Chol50) (c) versus the corresponding fitted models. Red points refer to MD simulations, continuous blue lines to the best-fit models. Black dashed lines refer to the asymptotic Brownian regime. Vertical orange, cyan and green dashed lines indicate respectively $1/\omega_s$, $1/\omega_0$ and τ.

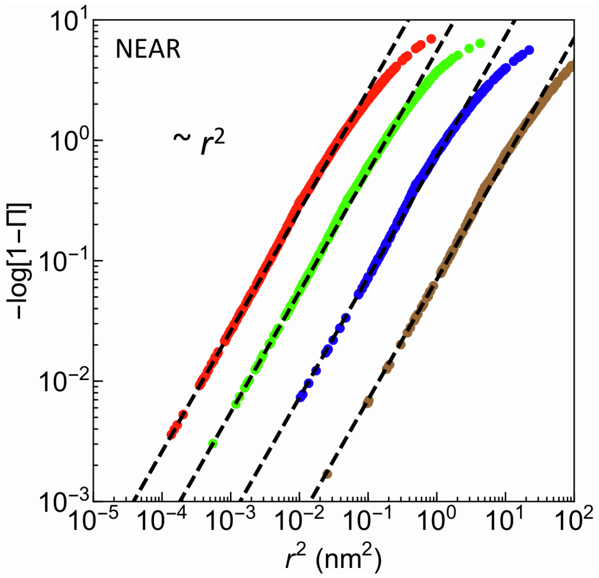

(a)

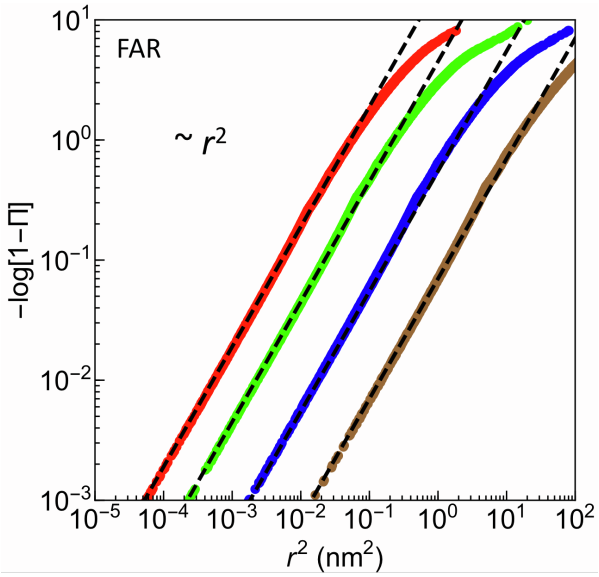

(b)

FIG. 11: Cumulative distribution $\Pi(r^2, \Delta)$ for NEAR (a) and FAR (b) lipids in the mixed membrane with $\Delta = 1, 10, 100, 1000$ ns (from left to right). The dashed lines correspond to theoretical curves proportional to r^2.

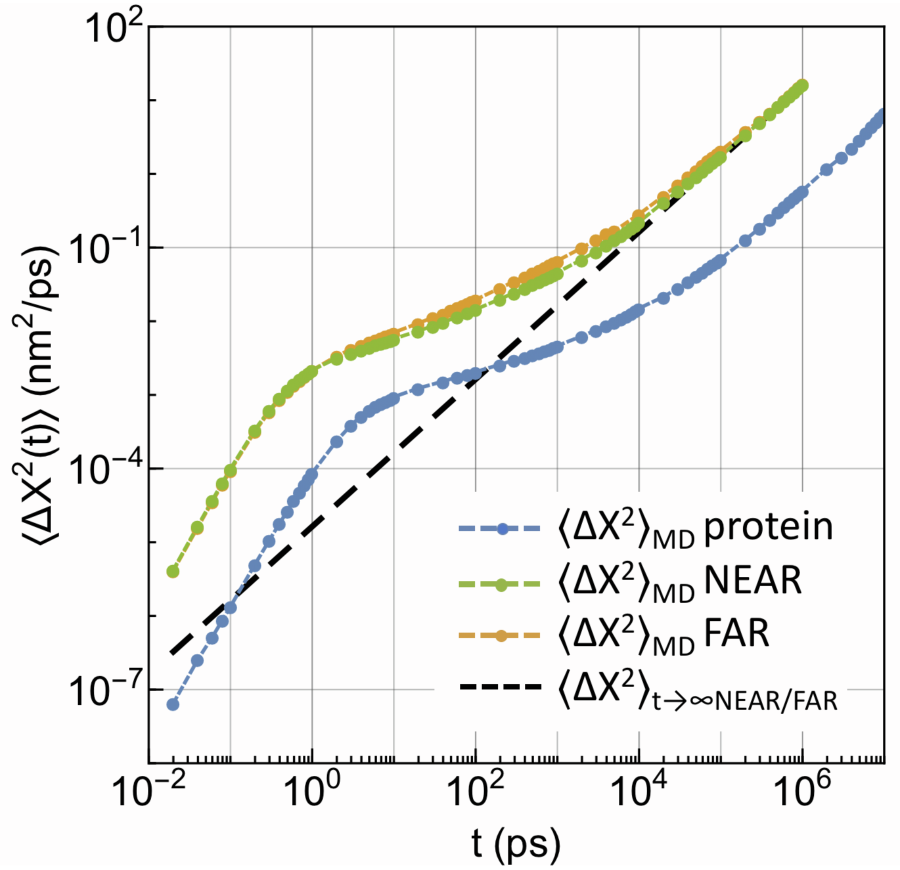

FIG. 12: MSD data from MD simulations of NEAR/FAR lipids. For comparison the MSD of the protein is shown as well. Black dashed line refers to the asymptotic Brownian regime of the lipids (not distinguishable between NEAR/FAR).

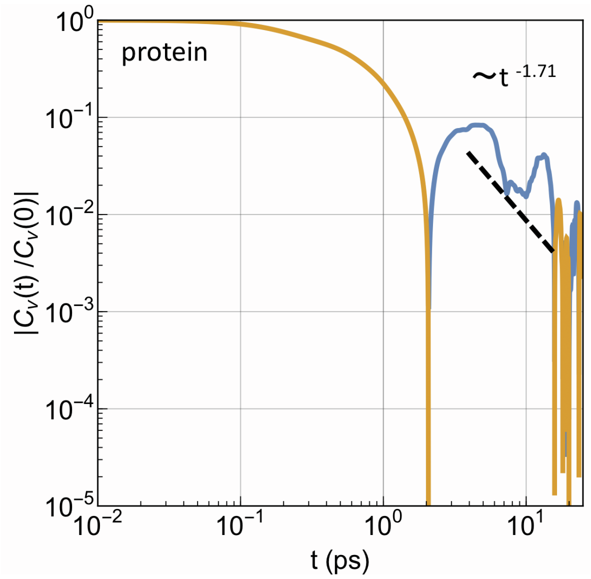

(a)

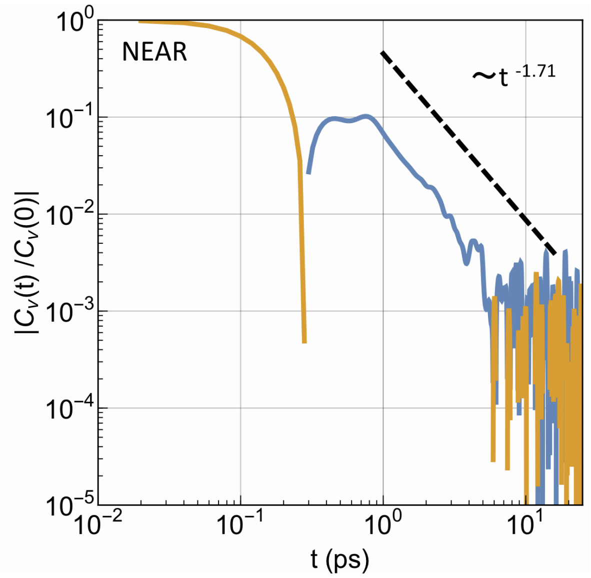

(b)

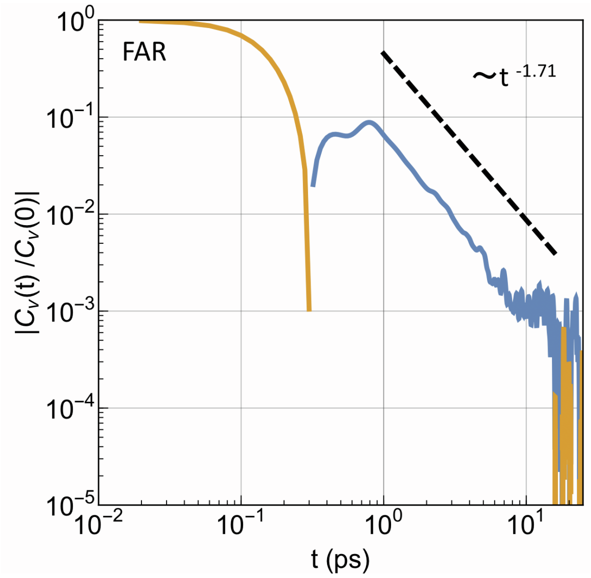

(c)

FIG. 13: Absolute VACFs of protein (a), NEAR (b) and FAR (c) lipids as obtained from MD simulations. Dark yellow and blue indicate respectively positive and negative correlations. The asymptotic behavior of the protein VACF as predicted by the model is shown as black dashed line for comparison.
